# Integrated metabolomic and transcriptomic analysis reveals the regulatory mechanisms of flavonoid and alkaloid biosynthesis in the new and old leaves of *Murraya tetramera* Huang

**DOI:** 10.1186/s12870-024-05066-9

**Published:** 2024-06-05

**Authors:** Tao Zhou, Qinqin Xing, Jiahao Bu, Wenjun Han, Zhiguo Shen

**Affiliations:** 1 College of Life Science and Technology, Central South University of Forestry and Technology, No.498, South Shaoshan Road, Changsha, 410004 Hunan Province China; 2 Henan Academy of Forestry, Zhengzhou, 450008 Henan Province China

**Keywords:** *M. Tetramera*, Flavonoids, Alkaloids, Metabolome and transcriptome

## Abstract

**Background:**

*Murraya tetramera* Huang is a traditional Chinese woody medicine. Its leaves contain flavonoids, alkaloids, and other active compounds, which have anti-inflammatory and analgesic effects, as well as hypoglycemic and lipid-lowering effects, and anti-tumor effects. There are significant differences in the content of flavonoids and alkaloids in leaves during different growth cycles, but the synthesis mechanism is still unclear.

**Results:**

In April 2021, new leaves (one month old) and old leaves (one and a half years old) of *M. tetramera* were used as experimental materials to systematically analyze the changes in differentially expressed genes (DEGs) and differentially accumulated metabolites (DAMs) with transcriptomics and metabolomics technology. This was done to identify the signaling pathways of flavonoid and alkaloid synthesis. The results showed that the contents of total alkaloids and flavonoids in old leaves were significantly higher than those in new leaves. Thirteen flavonoid compounds, three isoflavone compounds, and nineteen alkaloid compounds were identified, and 125 and 48 DEGs related to flavonoid and alkaloid synthesis were found, respectively. By constructing the KEGG (Kyoto Encyclopedia of Genes and Genomes) network of DEGs and DAMs, it was shown that the molecular mechanism of flavonoid biosynthesis in *M. tetramera* mainly focuses on the “flavonoid biosynthetic pathway” and the “flavonoid and flavonol biosynthetic pathway”. Among them, p-Coumaryl alcohol, Sinapyl alcohol, Phloretin, and Isoquercitrin were significantly accumulated in old leaves, the up-regulated expression of CCR (cinnamoyl-CoA reductase) might promote the accumulation of p-Coumaryl alcohol, upregulation of F5H (ferulate-5-hydroxylase) might promote Sinapyl alcohol accumulation. Alkaloids, including indole alkaloids, pyridine alkaloids, imidazole alkaloids, and quinoline alkaloids, were significantly accumulated in old leaves, and a total of 29 genes were associated with these substances.

**Conclusions:**

These data are helpful to better understand the biosynthesis of flavonoids and alkaloids in *M. tetramera* and provide a scientific basis for the development of medicinal components in *M. tetramera*.

**Supplementary Information:**

The online version contains supplementary material available at 10.1186/s12870-024-05066-9.

## Background

*Murraya tetramera* Huang is a small evergreen tree of the genus *Murraya* in Rutaceae, mainly distributed in Guangxi and Yunnan Provinces in China [1]. It has important medicinal, edible and ornamental value, and its leaves are the main medicinal part of the plant, which can be harvested throughout the year. It is commonly used in the treatment of cold, fever, bronchitis, asthma, rheumatism, numbness, muscle and bone pain, falling stasis, swelling and pain, stomachache and edema, etc [2]. Therefore, the leaves of *M. tetramera* have a high demand and market development potential in the field of medicine and the development of health tea in China. However, due to the over-exploitation of plant resources, the natural resources of *M. tetramera* are in danger. With the rise of artificial cultivation technology in the application of Chinese herbal medicine, the dilemma of the lack of resources for many medicinal plants has been solved, but the medicinal ingredients contained in most of the artificial plants are far less valuable than those in wild habitats, and the current situation of the artificial cultivation of *M. tetramera* is also the same. Therefore, revealing the accumulation regulation mechanism of plant metabolites will help to improve the scientific cultivation system of artificial medicinal plants, thereby further improving the medicinal value of artificial plants.

The medicinal ingredients of plants are mainly reflected in the types and cumulative content of secondary metabolites, which are usually produced by plants in response to biotic and abiotic stresses of the environment. These include bioactive substances such as phenols, terpenes, alkaloids, saponins, glycosides, and polysaccharides, which play important roles in antioxidant, anti-inflammatory, anti-cancer, and immunomodulation [3]. Flavonoid compounds are the main phenolic compounds in plant metabolites, mainly including flavanones, flavanols, flavones, and flavonols and so on [4], which play an important role in the agronomic and nutritional value of plants, affecting the ornamental and economic value of plants [5–7]. In addition, flavonoid compounds can scavenge free radicals, participate in the regulation of the cell cycle, induce apoptosis and autophagy, and significantly inhibit the proliferation and invasion of cancer cells [8]. Plant alkaloids, naturally occurring nitrogenous compounds found in about 20% of plant species, can be categorized into benzylisoquinoline alkaloids, monoterpenoid indole alkaloids, tropane alkaloids, etc. based on the carbon backbone structure of the compounds. These compounds are significant for defending against plant diseases and pests and are also active components in various clinical drugs. Alkaloids have been used in human medicine for 3,000 years, for treating diarrhea, fever, and snake venoms, as well as serving as astringents and sedatives, containing plant-derived alkaloids or synthetic analogues as their main active ingredients [9]. Currently, about 12,000 kinds of animal and plant alkaloids have been developed for use in drugs, stimulants, anesthetics, and other products for pain relief, anti-proliferation, cancer treatment, and so on [10, 11].

Previous studies have shown that the plants of the genus *Murraya* are rich in active substances such as flavonoids and carbazole alkaloids. With the application and development of detection technology, the effective components with medicinal value have been gradually isolated and identified. In vitro activity studies have shown that the flavonoids and alkaloids of *M. tetramera* have significant inhibitory effects on many cancer cells [12, 13], as well as anti-inflammatory and analgesic effects [14, 15]and anthelmintic effects [16]. Alkaloids are intermediates of plant nitrogen metabolism derived from different amino acids. Flavonoids are 15-carbon compounds with two aromatic rings connected by three-carbon bridges. One aromatic ring and the bridge are converted from phenylalanine, and the other aromatic ring is derived from the malonic acid pathway. This means that alternative pathways such as glycolysis, the citric acid cycle, the pentose phosphate pathway, and the tricarboxylic acid cycle all have significant effects on the accumulation of alkaloids and flavonoids [17]. There are also significant differences in flavonoids and alkaloids in the same tissue of plants at different growth stages. For example, the difference in the ability of young leaves and old leaves of *Arabidopsis thaliana* to resist some abiotic stresses is caused by the difference in secondary metabolites [18]. The differential accumulation of metabolites in various habitats and tissues of plants is closely related to the expression of enzymes and regulatory transcription factors in various metabolic pathways. For example, in the study of the transcription metabolism of *Ginkgo biloba*, GbbHLH91 was a transcription factor isolated from the bHLH transcription factor family, which had a significantly positive regulatory effect on the synthesis of flavonoids in its leaves. This transcription factor could positively regulate the expression of related enzymes in the process of flavonoid synthesis by co-expression with the MYB transcription factor [19]. In *Dendrobium huoshanense*, 124 and 58 genes related to leaf and root were identified to regulate alkaloid synthesis, and five key enzyme genes in the key terpenoid pathway were further screened for fluorescence quantitative verification. The results have laid a foundation for the study of the accumulation mechanism of medicinal substances of *Dendrobium huoshanense* [20]. Therefore, the elucidation of the accumulation mechanism of secondary metabolites in medicinal plants has an important impact on the further development of their medicinal value [10, 11].

Leaves are the main medicinal parts of *M. tetramera*, but the accumulation characteristics and mechanism of medicinal active substances in leaves have not yet been reported, which seriously restricts the development and utilization of this medicinal plant. Therefore, in this study, new (one month old) and old (one and a half years old) leaves of *M. tetramera* were selected for extensive metabolomic and transcriptomic analyses, focusing on the flavonoid and alkaloid biosynthesis pathways. A total of 13 flavonoid compounds and 19 alkaloid compounds were identified to be significantly different between the new and old leaves of *M. tetramera*. A total of 125 genes related to flavonoid biosynthesis and 48 genes related to alkaloid biosynthesis were found to be differentially expressed, and the specific DEGs expression levels were verified by RT-qPCR. This data will help to better understand the biosynthesis of flavonoids and alkaloids in *M. tetramera*, construct the biosynthetic pathway of flavonoids and alkaloids in *M. tetramera*, and identify the key regulatory genes of flavonoids and alkaloids biosynthetic pathway, which will lay a foundation for further revealing the biosynthetic metabolic pathway, accumulation mechanism and plant stress resistance of medicinal components of *M. tetramera*.

## Results

### Determination of flavonoids and alkaloids in new and old leaves of *M. tetramera*

**Fig. 1:**
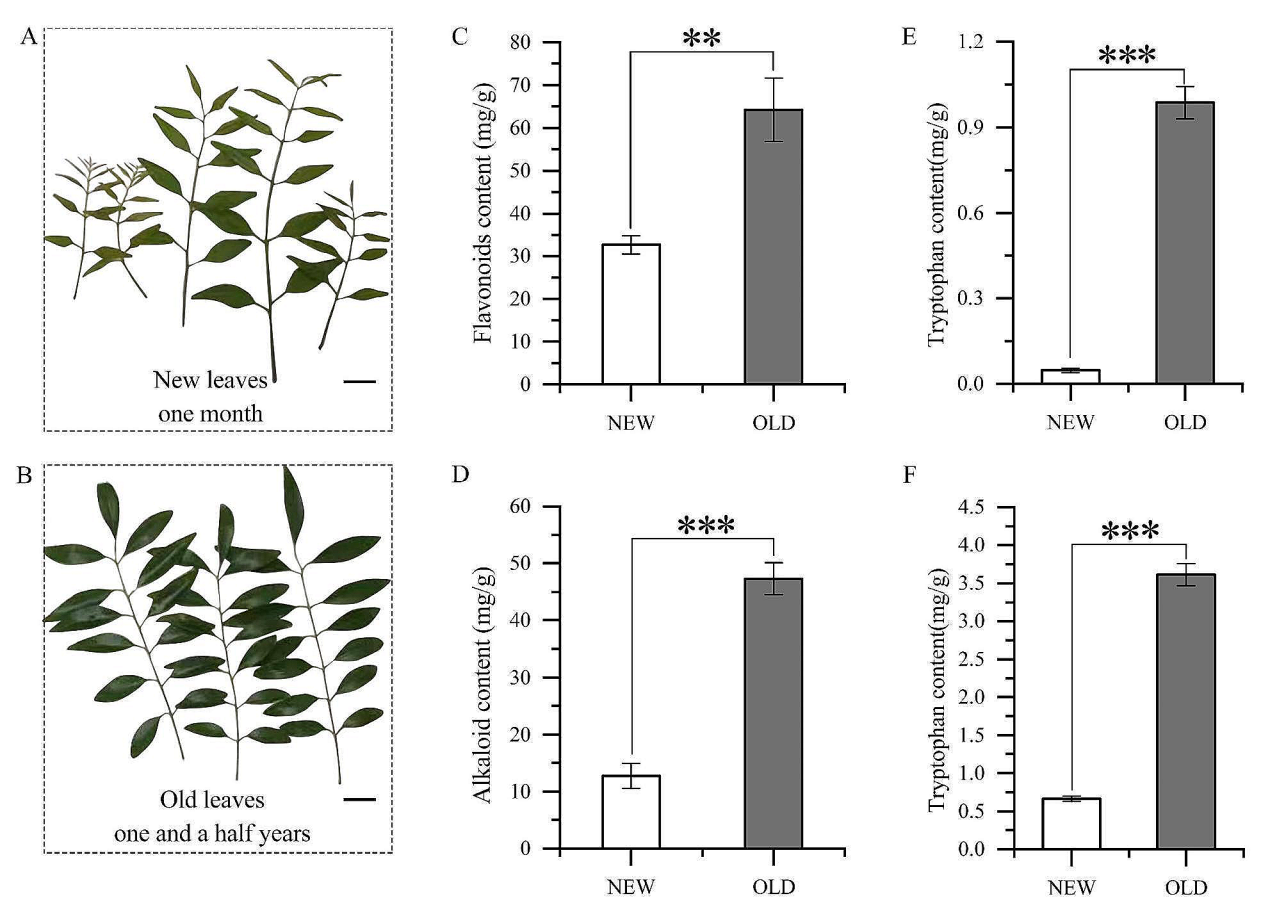
Phenotype and metabolite content determination of *M. tetramera*. **A** New leaves of *M. tetramera*, Bars = 1 cm. **B** Old leaves of *M. tetramera*, Bars = 1 cm. **C** Total flavonoid content in new and old leaves of *M. tetramera*. **D** Total alkaloid content in new and old leaves of *M. tetramera*. **E** Tyrosine content in new and old leaves of *M. tetramera*. **F** Tryptophan content in new and old leaves of *M. tetramera*. The experimental data above indicate the mean ± standard error (*n* = 3), *p < 0.05, **p < 0.01, ***p < 0.001 (student’s t-test)

Two-year-old *M. tetramera* plants was selected and sampled according to the growth cycle difference of the leaf parts. The leaves with a one-month growth cycle at the top were taken as the new leaves (Fig. 1A and B). The contents of total alkaloids and flavonoids in the old leaves (OLD) were significantly higher than those in the new leaves (NEW), by 272.6% (*P* < 0.01) and 196.8% (*P* < 0.001) (Fig. 1C and D), respectively. This result indicated that a large number of alkaloids and flavonoids accumulate in the leaves of *M. tetramera* with the continuation of the growth cycle, and the accumulation of alkaloids and flavonoids does not affect the evergreen growth characteristics of the leaves. In addition, the contents of aromatic amino acids related to alkaloid synthesis were determined. Tyrosine and tryptophan accumulated significantly in old leaves, being 2041.3% (*P* < 0.001) and 442.9% (*P* < 0.001) higher than that in new leaves, respectively (Fig. 1E and F). The results indicated that there are likely key genes and transcription factors that significantly regulate alkaloid synthesis in old leaves.

### Metabolome PCA and OPLS-DA analysis

**Fig. 2:**
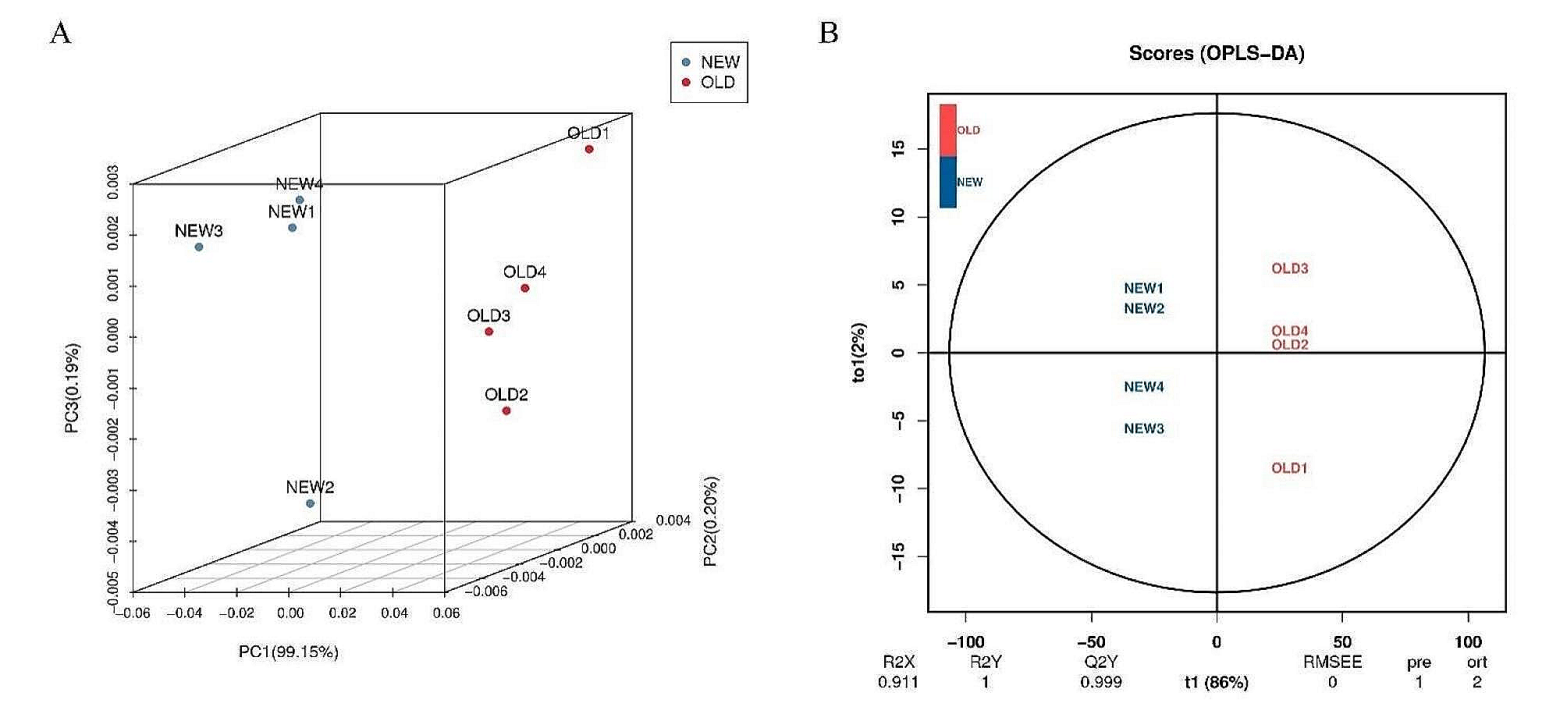
PCA analysis and OPLS-DA score plot for all samples. **A** PCA (Principal component analysis) clustering based on metabolome data. **B** OPLS-DA score plot based on metabolome data

Metabolome analysis for NEW and OLD was conducted using the UPLC-MS/MS detection platform, alongside a plant metabolism database and multivariate statistical analysis, developed by BioMarker Biotechnology Co., Ltd. (Beijing, China). The results of Principal component analysis (PCA) showed a high degree of dispersion between the NEW and OLD groups, with obvious metabolic differences (Fig. 2A). The fitting degree of orthogonal projections to latent structure-discriminant analysis (OPLS-DA) for the independent variable X and the dependent variable Y is R2X = 0.911, R2Y = 1, and the predictive degree Q2Y = 0.999, respectively (Fig. 2B). The OPLS-DA model is an effective model, and the sample data have high reliability, stability and predictability, this indicates the presence of significantly different types and contents of metabolites in the new and old leaves of *M. tetramera*.

### DAMs screening and KEGG enrichment analysis

**Fig. 3:**
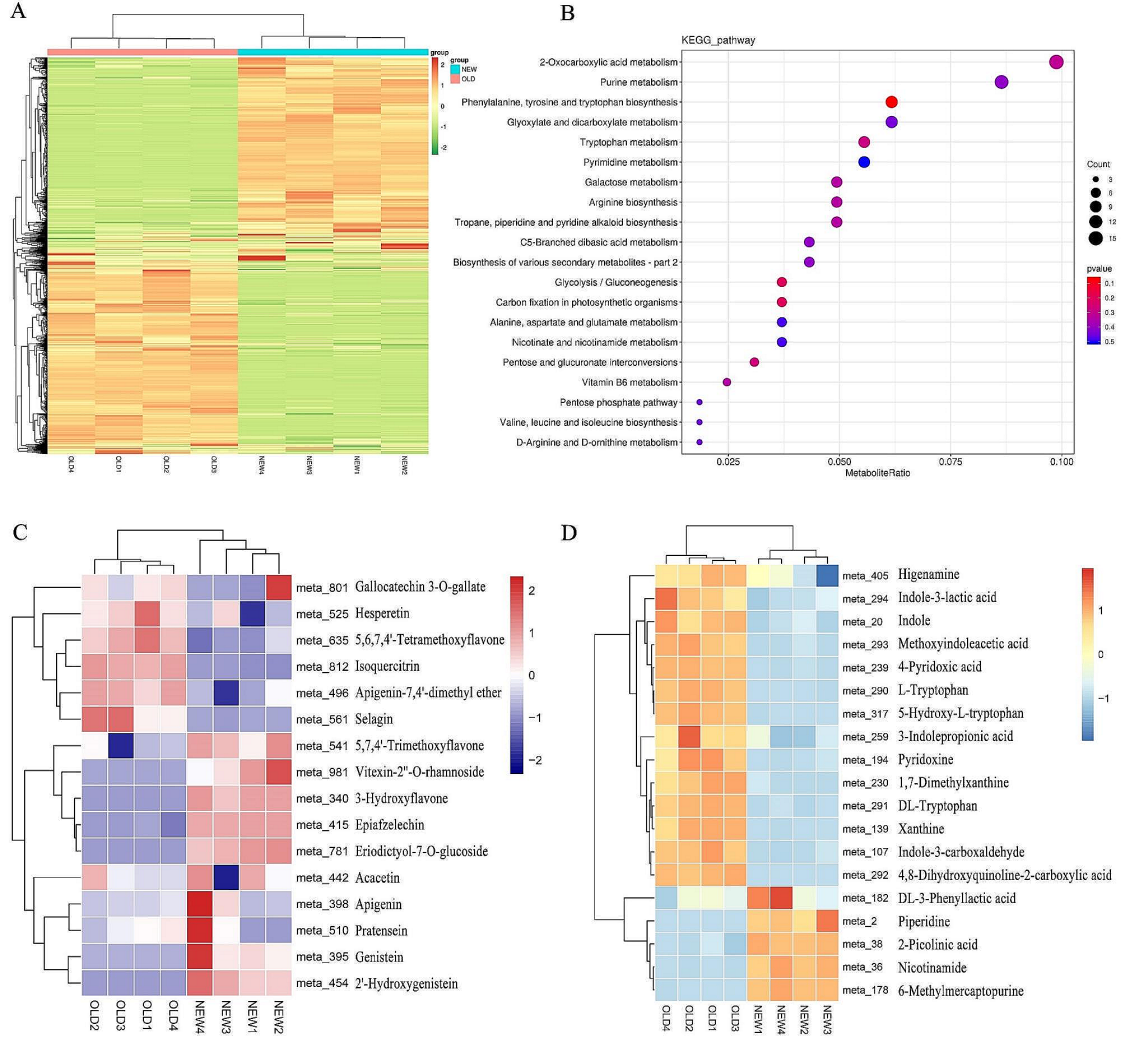
Clustering and enrichment map of DAMs. **A** Clustering of DAMs in new and old leaves of *M. tetramera*. The horizontal axis represents the sample name, and the vertical axis represents clustered metabolites; warm colors indicate substances with high content, and cold colors indicate substances with low content. **B** KEGG pathway enrichment of DAMs between new and old leaves of *M. tetramera*. The abscissa is the sample name, the ordinate is the KEGG pathway name, the number of metabolites enriched in this pathway in the size of the circle, and the pvalue is marked in the color. **C** The Heat map of flavonoid metabolites in new and old leaves of *M. tetramera*. **D** The Heat map of alkaloid metabolites in new and old leaves of *M. tetramera*. Meta is the ID of different metabolites, the abscissa is the sample name, the ordinate is the metabolite, and the color difference represents the difference in metabolite content

In comparison with NEW, 850 DAMs identified in OLD showed that 427 had higher levels in OLD and 425 had higher levels in NEW. (Fig. 3A). 310 DAMs were annotated by the KEGG database, and KEGG enrichment analysis showed that the DAMs between NEW and OLD were mainly enriched in 2-Oxocarboxylic acid metabolism (ko01210), Purine metabolism (ko00230), Phenylalanine tyrosine and tryptophan biosynthesis (ko00400), Glyoxylate and dicarboxylate metabolism(ko00630), Tryptophan metabolism(ko00380) and Pyrimidine metabolism(ko00240) (Fig. 3B). The DAMs were grouped into 41 categories. The most abundant categories included Carboxylic acids and derivatives, Fatty Acyls, Organooxygen compounds, Benzene and substituted derivatives, and Organonitrogen compounds (Table S1). Thirteen metabolites were related to flavonoids (meta_340; meta_398; meta_415; meta_442; meta_496; meta_525; meta_541; meta_561; meta_635; meta_781; meta_801; meta_812; meta_981), and three to isoflavones (meta_395; meta_454; meta_510), in which 10 metabolites were higher in NEW than in OLD and 6 metabolites were higher in OLD than in NEW (Fig. 3C). There were 19 metabolites related to alkaloids, including indole alkaloids (meta_20; meta_107; meta_259; meta_290; meta_291; meta_293; meta_294; meta_317), imidazole alkaloids (meta_139; meta_178; meta_230), quinoline alkaloids (meta_292), isoquinoline alkaloids (meta_405), pyridine alkaloids (meta_36; meta_38; meta_194; meta_239), piperidines (meta_2) and scopolamine alkaloids (meta_182), among which 5 metabolites were higher in NEW than in OLD and 14 metabolites were higher in OLD than in NEW (Fig. 3D). The difference in these secondary metabolites suggests one of the molecular mechanisms that the old leaves and the new leaves of *M. tetramera* have different medicinal values.

### Transcriptome assembly and functional annotation classification

In this study, six cDNA libraries were constructed, including three from new leaves (NEW-1, NEW-2, NEW-3) and three from old leaves (OLD-1, OLD-2, OLD-3). Transcriptome sequencing yielded a total of 44.35 Gb of clean data, with each sample contributing approximately 7.33 Gb. An average of 24,725,123 clean reads were obtained by new leaves sequencing and the old leaves sequencing obtained an average of 24,693,488 clean reads, each transcript contained 44.38–44.72% GC. The average GC content was 44.52%, and the Q30 base percentage was 94.03% or more, indicating that the sequencing quality was good and could be used for de novo assembly and expression analysis (Table S2). The reads were assembled and quantified using Trinity, and a total of 38,989 unigenes were obtained. Among them, 15,147 unigenes were more than 1 kb in length, the N50 of unigenes was 2,420, and the average length of unigenes was 1,328.14, indicating high assembly integrity (Table S3). To understand the overall expression pattern of the transcriptome, functional annotation of the assembled unigenes was performed from a systems biology perspective. Functional annotation revealed that 26,587 (68.19%) unigenes closely matched sequences in public protein databases, such as NR, Swiss-Prot, KEGG, COG, KOG, GO, and Pfam. The annotation results showed that 25,551 (80.36%) unigenes had significant similarity to protein sequences in the Nr database at the cutoff of E ≤ 1e-05, and 15,358 (39.39%) unigenes annotated by the SwissProt database (Table S4).

### GO classification and KEGG pathway analysis of DEGs

**Fig. 4:**
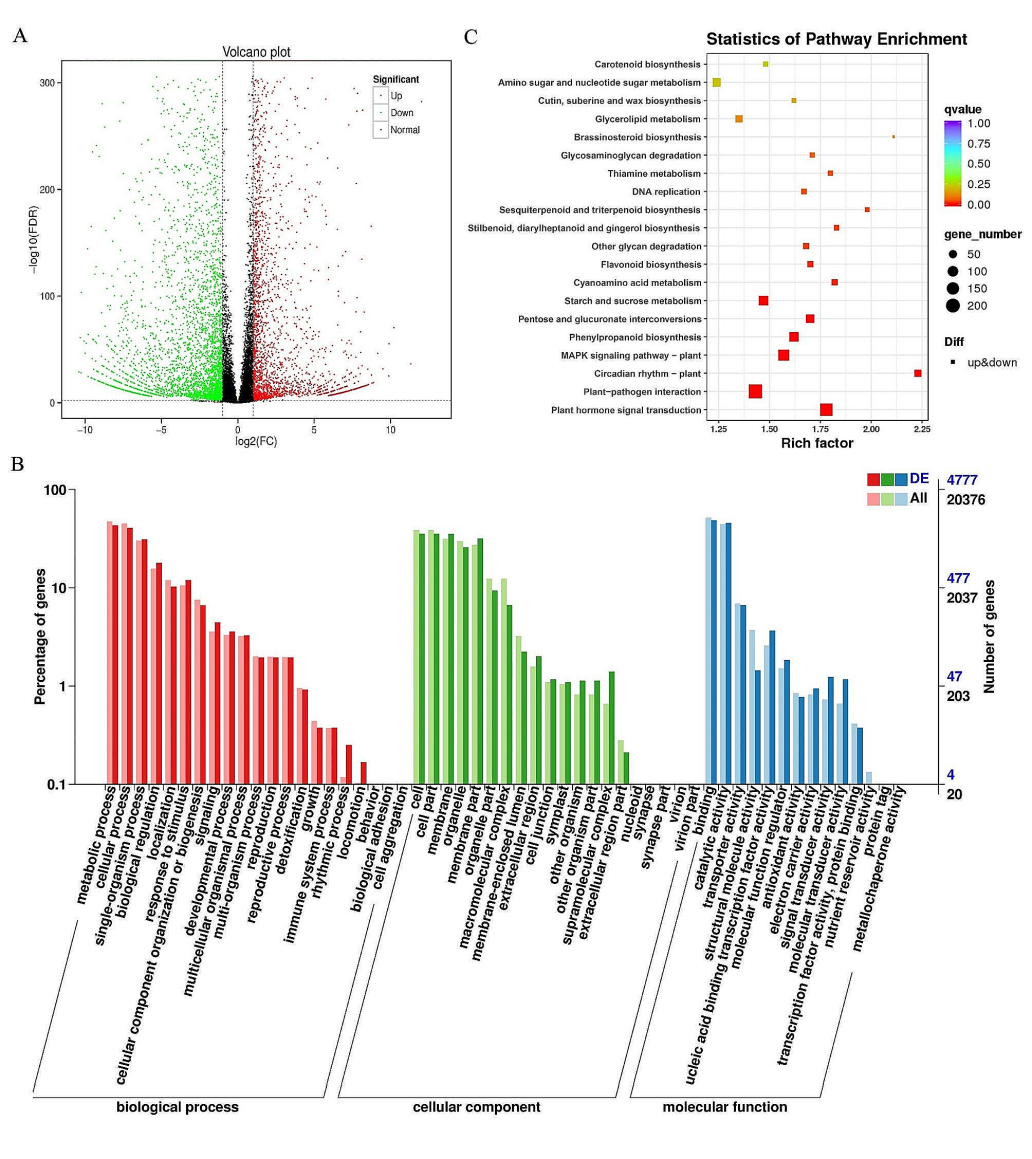
Analysis of DEGs. **A** volcano map of DEGs between new and old leaves of *M. tetramera*. **B** GO functional annotation of DEGs between new and old leaves of *M. tetramera*. **C** KEGG enrichment map of DEGs between new and old leaves of *M. tetramera*

Transcriptome data analysis showed that 13,081 genes were shared between NEW and OLD samples, with 2,086 genes unique to NEW and 1,362 genes unique to OLD. In comparison to NEW samples, 6,262 DEGs were identified in OLD samples, with 2,702 genes up-regulated and 3,560 genes down-regulated. (Fig. 4A). Of the 6,262 DEGs, 5,845 genes were annotated. Specifically, 4,777 genes were classified under GO categories, and 3,871 genes were associated with KEGG pathways, 1,814 genes were annotated in the COG classification, and 4,865 genes were enriched in the eggNOG pathway. Analysis of DEGs annotated into GO functional categories (Table S5), BLAST2GO grouped a total of 4,777 annotated unigenes into 63 functional categories. Among these groups, 20 were involved in ‘biological processes’ (BP), 14 in ‘cellular components’ (CC), and 21 in ‘molecular functions’ (MF). In the functional classification of biological process, the most DEGs were annotated to metabolic process, followed by cellular process and single organic process. In the cellular component functional classification, most genes were annotated to cell and cell part. In the molecular function classification, the most DEGs were annotated to binding and catalytic activity (Fig. 4B). In the COG functional classification, the three top terms were G (Carbohydrate transport and metabolism), T (Signal transduction mechanisms) and R (General function prediction only).

Using KEGG, 1,971 DEGs were classified into 133 metabolic pathways. The pathway were divided into five branches: Cellular Processes, Environmental Information Processing, Genetic Information Processing, Metabolism, and Organismal Systems (Fig. S1). The top 20 KEGG enrichment pathways were shown in Fig. 4C, and the most significant pathways were Plant hormone signal transduction (ko04075), plant-pathogen interaction (ko04626), and Circadian rhythm-plant (ko04712). Combining gene annotations with KEGG pathway insights revealed 125 DEGs linked to flavonoid synthesis and 48 DEGs associated with alkaloid synthesis. Most of those DEGs were enriched in the binding and catalytic activity of molecular function in GO classification. In addition, in the 20 significantly enriched KEGG pathways, there were 40 DEGs related to flavonoid synthesis in the Stilbenoid, diarylheptanoid and gingerol biosynthesis (ko00945) pathway, 11 in Starch and sucrose metabolism (ko00500) pathway, 54 in Phenylpropanoid biosynthesis (ko00940) pathway, 8 in Flavonoid biosynthesis (ko00941) pathway, and 3 in Circadian rhythm-plant (ko04712) pathway. Two DEGs related to alkaloid synthesis were enriched in the Cyanoamino acid metabolism (ko00460) pathway.

**Fig. 5:**
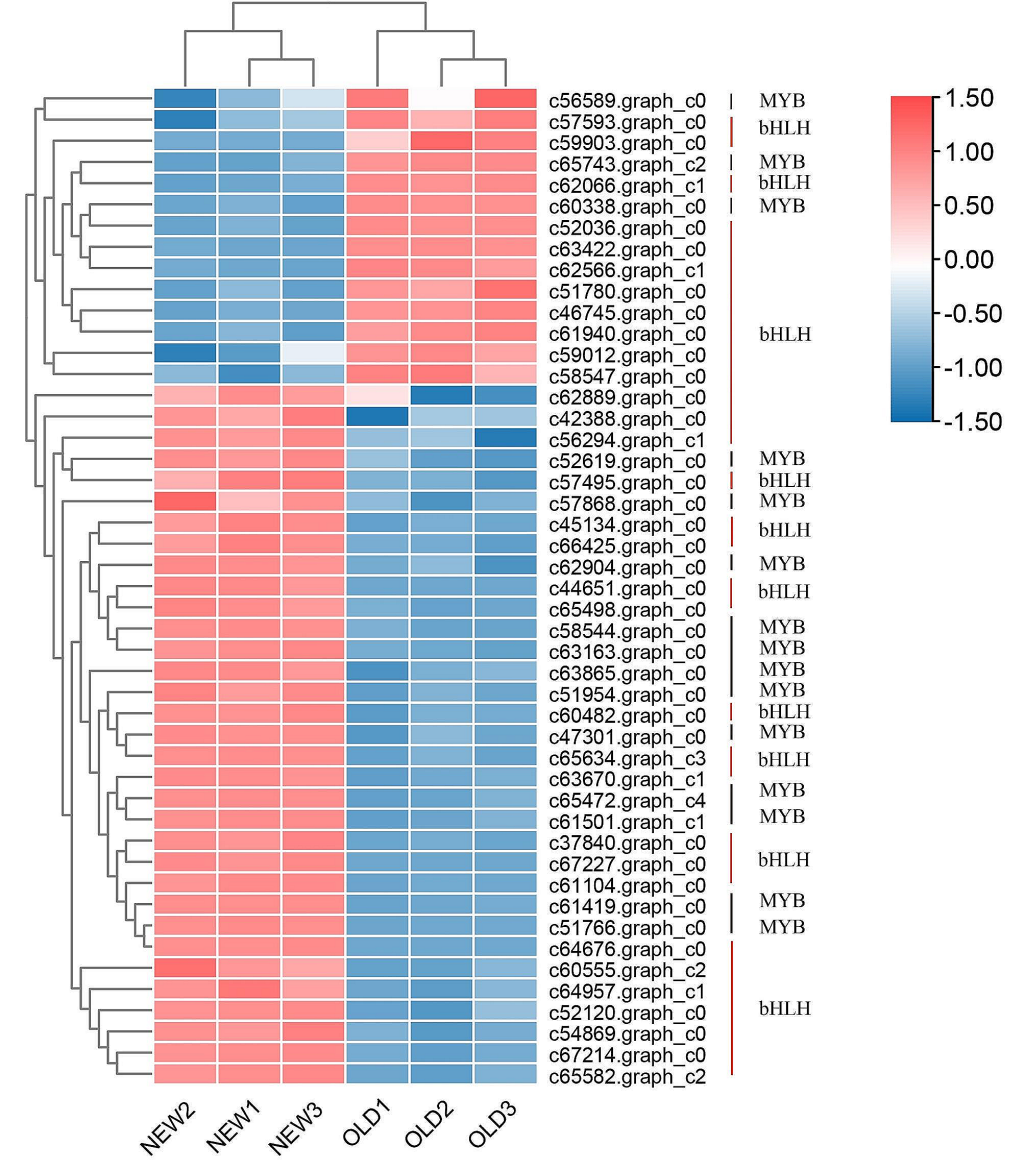
Heat map of expression of transcription factor genes related to flavonoids and alkaloids in new and old leaves of *M. tetramera*. The abscissa is the sample name, the color difference represents the expression level of each gene, warm colors represent high expression, and cold colors represent a low expression

Transcription factors (TFs) play an important role in regulating the activity of flavonoids and alkaloids biosynthesis, along with other secondary metabolism pathways. A total of 1,785 genes were identified as transcription factors, The most abundant TF family was C2H2 TF, including 489 unigenes. The C2H2 TF family is one of the largest family of transcription factors in plants, regulating many biological processes such as plant morphogenesis, transcriptional activation, and stress. Transcription factors related to the regulation of plant flavonoid compounds and alkaloids include MYB, bHLH, ERF, and WRYK [6, 21, 22]. In this plant, two transcription factor families showed differential expression: MYB and bHLH. Among these TFs, 15 DEGs were annotated as MYB transcription factors, with 3 up-regulated and 12 down-regulated in the NEW-vs-OLD comparison. Similarly, 32 DEGs were annotated as bHLH transcription factors, with 11 up-regulated and 21 down-regulated in old leaves (Fig. 5), indicating that these differentially expressed unigenes in MYB and bHLH transcription factor families might be associated with leaf growth stages of *M. tetramera*. Combining these findings with the comparative analysis of total flavonoids and alkaloids in new and old leaves, it can be inferred that the up-regulated transcription factors in old leaves may significantly influence the synthesis of flavonoids and alkaloids.

### Integrated analysis of metabolomic and transcriptomic data

**Fig. 6:**
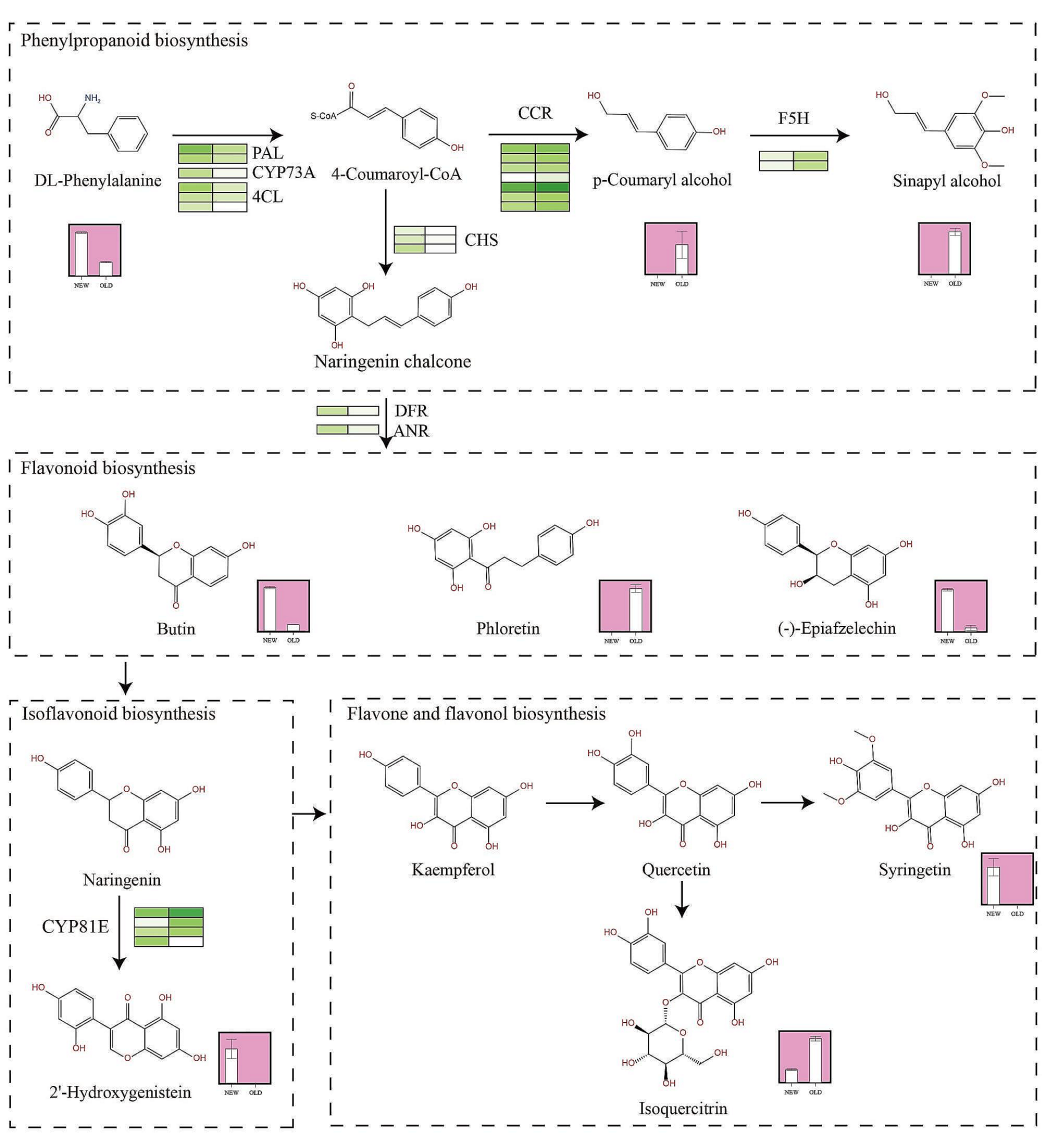
Biosynthetic and accumulation pathways of flavonoid compounds in new and old leaves of *M. tetramera*. Green represents the expression heat map of the corresponding gene of the related enzyme in the metabolic pathway, with darker green indicating higher expression. The pink box represents the content of the metabolite in the new and old leaves, and an arrow $(\rightarrow)$ indicates positive regulation. PAL: phenylalanine ammonia-lyase; CYP73A: trans-cinnamate 4-monooxygenase; 4CL:4-coumarate–CoA ligase; CCR: cinnamoyl-CoA reductase; F5H: ferulate-5-hydroxylase; CHS: chalcone synthase; DFR: bifunctional dihydroflavonol 4-reductase; ANR: anthocyanidin reductase; CYP81E: 4’-methoxyisoflavone 2’-hydroxylase

Through the integrated analysis of the metabolome and transcriptome of *M. tetramera*, 81 pathways were simultaneously annotated with differential metabolites and genes. Pathways with a p-value < 0.01 include Cyanoamino acid metabolism, Flavone and flavonol biosynthesis, Pentose and glucuronate Interremodeling, Phenylpropanoid biosynthesis, Plant hormone signal transduction, Starch and sucrose metabolism, and Thiamine metabolism (Fig. S2, Table S6). Significantly enriched metabolites related to flavonoid biosynthesis in the *M. tetramera* metabolome correlated with related DEGs (Fig. 6). In the flavonoid precursor synthesis pathway, 85 DEGs and 11 DAMs in the NEW-VS-OLD comparison were enriched in the Phenylpropanoid biosynthesis (ko00940) pathway. This pathway, annotated for expressing enzymes such as phenylalanine ammonia-lyase (PAL) (c64871.graph_c0; c55211.graph_c0), trans-cinnamate 4-monooxygenase (CYP73A) (c50654.graph_c0), and 4-coumarate-CoA ligase (4CL) (c63945.graph_c2; c64871.graph_c2; c58307.graph_c0) showed downregulated. Seven homologous genes, noted as cinnamoyl-CoA reductase (CCR) genes, initiated positive regulation for the synthesis of p-Coumaryl alcohol((meta_137)). Two genes encoding for ferulate-5-hydroxylase (F5H) (c37991.graph_c0; c60994.graph_c1) were upregulated, positively influencing the synthesis of sinapyl alcohol (meta_306). There were 31 DEGs enriched in Flavonoid biosynthesis(ko00941), enzymes regulating product synthesis included chalcone synthase (CHS), dihydroflavonol 4-reductase (DFR), anthocyanidin reductase (ANR), phlorizin synthase (PGT1), where CHS (c51677.graph_c0; c61257.graph_c0; c67412.graph_c0) and DFR (c63011.graph_c0), significantly downregulated. ANR had two homologous genes, c62373.graph_c0 upregulated, and c67197.graph_c0 downregulated. The DAMs detected in this pathway include Butin(meta_408), Phloretin(meta_414), and Epiafzelechin(meta_415), had their synthesis positively regulated by the aforementioned enzymes. Among them, only Phloretin’s content in old leaves accumulated significantly, whereas the other substances were more abundant in new leaves than in old ones. Three isoflavones were identified as Genistein(meta_395), 2’-Hydroxygenistein(meta_454) and Pratensein(meta_510). Only 2’-Hydroxygenistein showed significantly higher content in new leaves compared to old leaves and was enriched in the Isoflavonoid Biosynthesis (ko00943) pathway, regulated by CYP81E (isoflavone/4’-methoxyisoflavone 2’-hydroxylase). Gene annotation revealed four homologous genes for this enzymes, one downregulated (c50564.graph_c0) and three upregulated (c64659.graph_c2; c37778.graph_c0; c47176.graph_c1). The DAMs enriched in the Flavone and flavonol biosynthesis(ko0094) pathway were Syringetin(meta_655), Isoquercitrin(meta_812), Apigenin(meta_398) and Acacetin(meta_442) (Fig. 6), among which Syringetin was positively regulated by the annotated gene anthocyanidin 3-O-glucosyltransferase (c57273.graph_c), which was significantly overexpressed in old leaves. It is worth noting that the contents of Phloretin and Isoquercitrin in old leaves were very significant (*P* < 0.01), being 28.05 times and 13.36 times of those in new leaves, respectively. Their accumulation might explain the higher total amount of flavonoids in old leaves. Secondly, through the relationship between the expression levels of metabolites and related regulatory enzyme genes, it was observed that the high content of flavonoids in new leaves might induce a feedback regulatory effect, resulting in low expression levels of related enzyme genes. This observation aligns with the correlation between the accumulation of metabolites in plants and the transcriptome under the natural growth cycle of green leafy plants. It helps maintain the leaf color in the evergreen state, preventing pigment imbalance caused by excessive accumulation of flavonoids. F5H and CCR were not regulated by the feedback of flavonoid accumulation in old leaves because these enzymes are also involved as limiting factors in the synthesis of sinapyl (S) monolignol in angiosperms. They play an important role in the synthesis of lignin, cellulose and polysaccharide substances in plants, resulting in some homologous genes maintaining continuous high expression with the growth of *M. tetramera* [23]. This indicates that the content of p-Coumaryl alcohol and sinapyl alcohol will continuously accumulate with the growth cycle of their leaves.

**Fig. 7:**
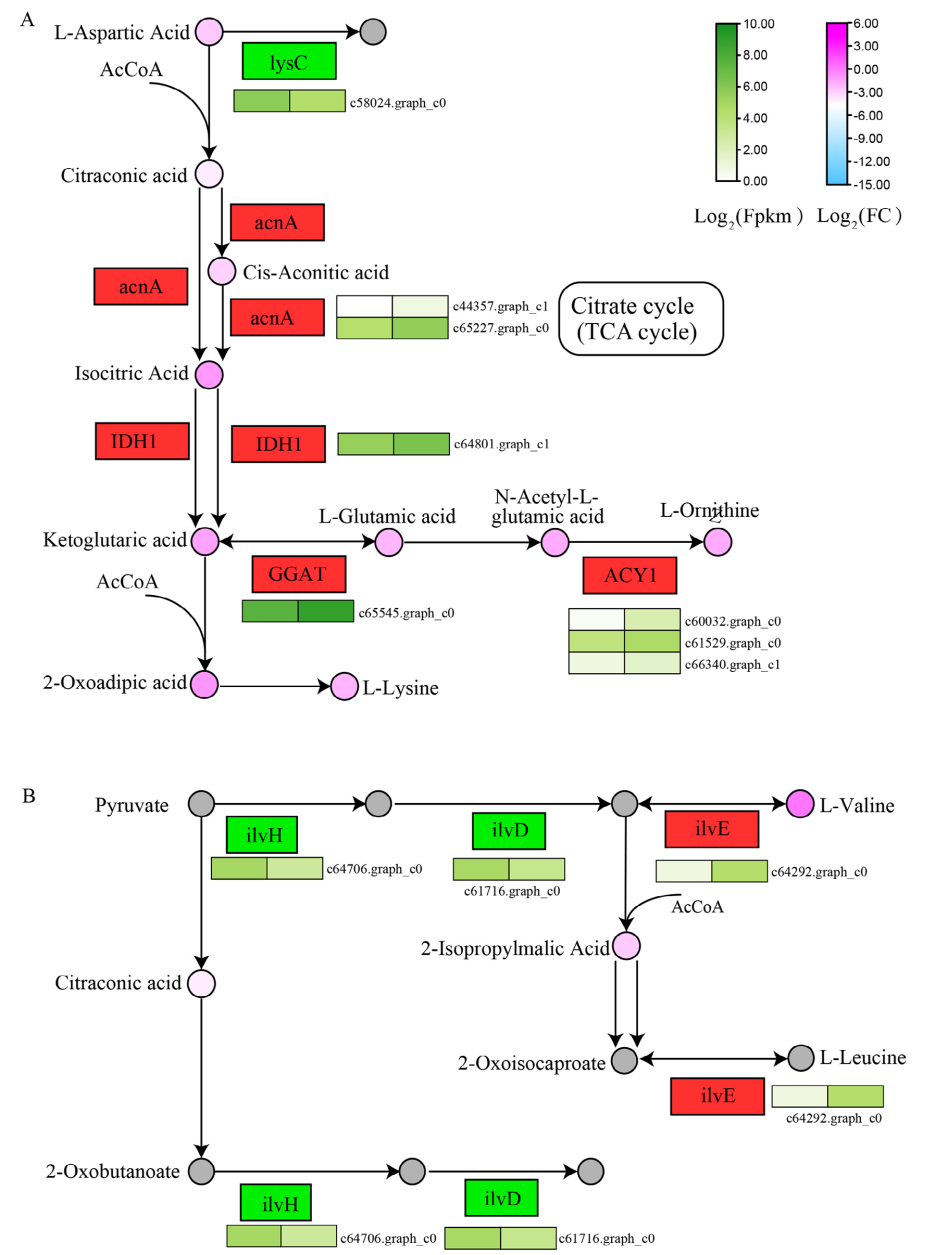
Pathways related to alkaloid synthesis in leaves of *M. tetramera*. **A** 2-Oxocarboxylic acid metabolism pathway. **B** Branched chain amino acid metabolism pathway. Circles represent metabolites, the darker the pink, the higher the content; the box represents the enzyme, the green represents the down-regulated gene expression, the red represents the up-regulated gene expression, and the heat map under the box represents the corresponding coding gene and expression heat map of the enzyme. lysC: aspartate kinase; acnA: aconitate hydratase; IDH1: isocitrate dehydrogenase; GGAT: glutamate–glyoxylate aminotransferase; ACY1: aminoacylase; ilvH: acetolactate synthase I/III small subunit; ilvD: dihydroxy-acid dehydratase; ilvE: branched-chain amino acid aminotransferase

Related DAMs and DEGs were significantly enriched in the pathways related to alkaloid synthesis, including 2-Oxocarboxylic acid metabolism(ko01210) and Isoquinoline alkaloid biosynthesis(ko00950), and Tropane, piperidine and pyridine alkaloid biosynthesis(ko00960). The 2-Oxocarboxylic acid metabolism pathway provides important precursors for alkaloid synthesis. Oxocarboxylic acid is a key intermediate for energy production and plays an important role in the synthesis of aromatic amino acids. This pathway includes Branched-chain amino acid metabolism. Branched-chain amino acids act not only as substrates for the synthesis of nitrogenous compounds, but also as signaling molecules that regulate glucose, fat, and protein metabolism through special signaling networks [24]. The key genes enriched in 2-Oxocarboxylic acid metabolism(ko01210), 1 ilvH(c64706.graph_c0), 1 ilvD(c61716.graph_c0) and 1 lysC(c58024.graph_c0) were significantly down-regulated, and 2 acnA(c65227.graph_c0; c44357.graph_c1), 1 IDH1(c64801.graph_c1), 1 GGAT(c65545.graph_c0), 3 ACY1(c60032.graph_c0; c66340.graph_c1; c61529.graph_c0) and 1 ilvE(c64292.graph_c0) were significantly up-regulated, 16 DAMs were enriched, of which 12 were higher in NEW than in OLD and 4 were higher in OLD than in NEW, the latter including L-Valine(meta_23), L-Tyrosine(meta_231), Citric Acid(meta_262), and L-Tryptophan(meta_290). The significantly up-regulated genes in this pathway indicated that the process of alkaloid synthesis in old leaves was more active, and precursor substances were consumed faster, such as L-Ornithine(meta_69), Isocitric Acid(meta_261), and α-Ketoglutaric acid(meta_112), containing less product, which might also be due to this reason (Fig. 7A). In the Branched-chain amino acid metabolism, the annotated ilVE gene significantly increased L-Valine content in old leaves, and the direct precursor of L-Valine was pyruvate in the glycolytic pathway. Acetohydroxy acid synthase (AHAS), acetoxy acid isomeroxidutase (AHAIR), dihydroxy acid dehydratase (DHAD) and transaminase (TA) were encoded by ilvBN, ilvC, ilvD and ilvE, respectively, under the catalysis of these enzymes, pyruvate finally synthesized L-Valine [25]. L-Valine may be the main source of amine precursors for the synthesis of alkaloids in *M. tetramera*. Meanwhile, the accumulation of L-Valine may feedback regulate the Branched-chain amino acid metabolism of old leaves, resulting in the down-regulation of ilvH and ilvD gene expression. Thus, the synthesis of some products is inhibited, for example, the detected content of 2-Isopropylmalic Acid is less in old leaves (Fig. 7B).

**Fig. 8:**
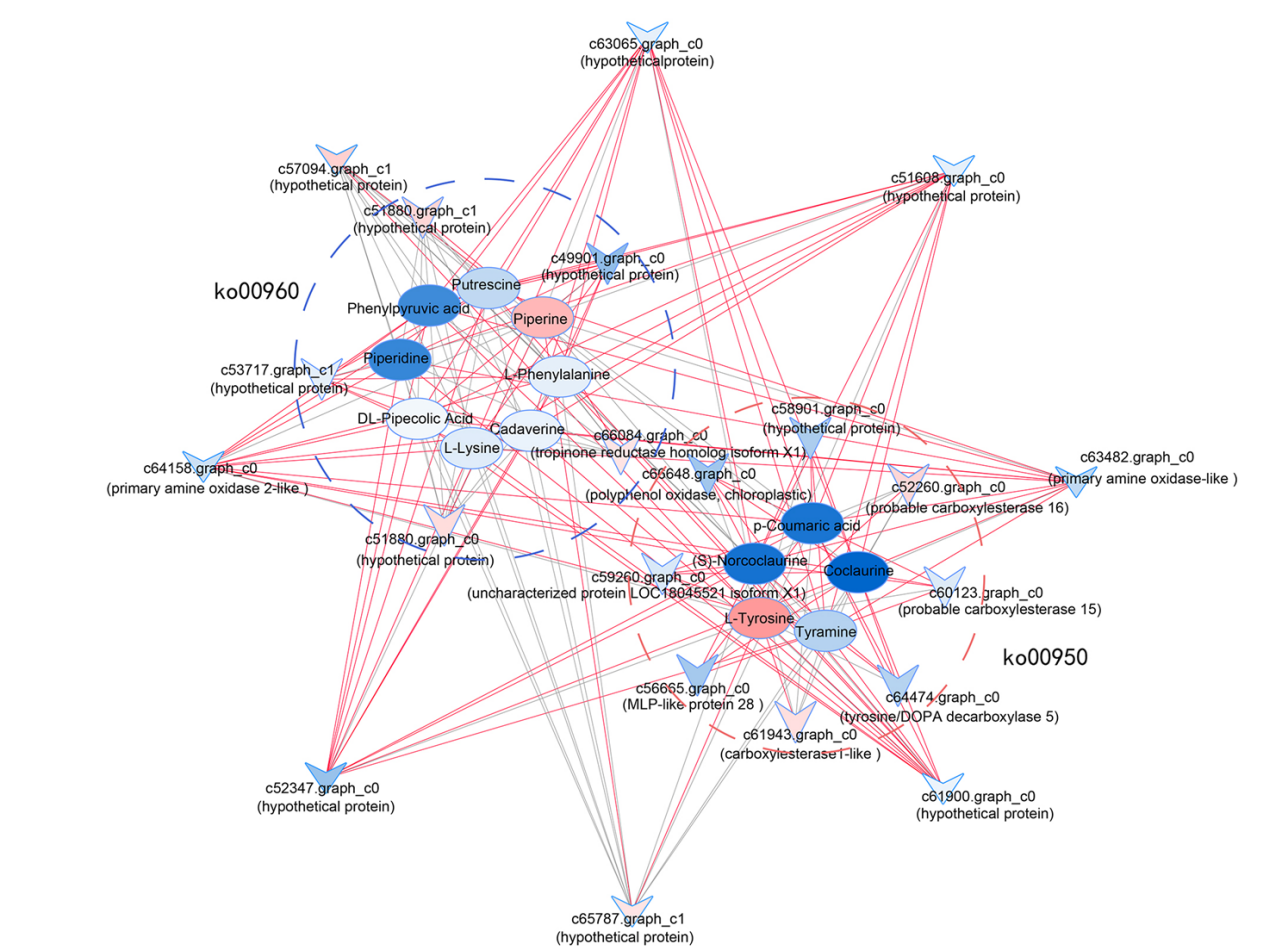
Correlation analysis between DEGs and differentially accumulated alkaloid metabolites in *M. tetramera* leaves. Arrows represent genes and ellipses represent metabolites. Red arrows represent NEW-vs-OLD genes that are up-regulated and blue ones that are down-regulated. The red ellipses represent the significant accumulation of NEW-vs-OLD metabolites and the blue represents the reduction of metabolites. The red lines represent the positive correlation between genes and metabolites, and the gray represents the negative correlation. Red circles represent ko00950 metabolic pathways, and blue circles represent ko00960 metabolic pathways

Due to the complexity of Isoquinoline alkaloid biosynthesis(ko00950), Tropane, piperidine and pyridine alkaloid biosynthesis(ko00960), we used the Pearson correlation algorithm to analyze the correlation between the metabolites measured and the annotated genes and then used Cytoscape to construct the network (Fig. 8). A total of 21 DEGs and 13 DAMs were enriched in the two pathways ko00950 and ko00960. In the isoquinoline alkaloid biosynthesis pathway, there were 8 unique genes and 5 metabolites. Among them, the expression of c59260.graph_c0, c56665.graph_c2, c60123.graph_c0, c66648.graph_c0, c58901.graph_c0, and c64474.graph_c0 has a positive regulatory relationship with the synthesis of p-Coumaric acid (meta_169), Tyramine (meta_200), (S)-Norcoclaurine (meta_404), and Coclaurine (meta_451). c56665.graph_c2 was annotated as MLP-like protein 28, c60123.graph_c0 was annotated as probable carboxylesterase 15, c66648.graph_c0 was annotated as polyphenol oxidase, chloroplastic, and c64474.graph_c0 was annotated as tyrosine/DOPA decarboxylase 5; c52260.graph_c2 and c61943.graph_c0 might have a positive regulatory relationship with the synthesis and accumulation of L-Tyrosine(meta_231). There were 5 unique genes and 8 metabolites in Tropane, piperidine and pyridine alkaloid biosynthesis pathway. The high expression of c51880.graph_c0, c51880.graph_c1, c66084.graph_c0 had a positive regulatory relationship with the synthesis and accumulation of Piperine(meta_453). In addition, there were 8 genes enriched on both pathways, among which, the high expression of c65787.graph_c1 and c57094.graph_c0 in old leaves was positively correlated with the accumulation of Piperine(meta_453) and L-Tyrosine(meta_231). Notably, L-Tyrosine and Piperine were significantly accumulated in old leaves, with their contents 19.18 and 9.85 times higher than those in new leaves, respectively. L-Tyrosine was most correlated with gene c52260.graph_c0, c61943.graph_c0, and c65787.graph_c0, c52260.graph_c0 was indicated as probable carboxylesterase 16, c61943.graph_c0 was annotated as carboxylesterase 1-like. And the positive regulation of Piperine was most correlated with the high expression of c65787.graph_c1 and c66084.graph_c0, they were annotated as tropinone reductase homolog isoform X1 and hypothetical protein, respectively.

### RT-qPCR was used to verify differential gene expression

**Fig. 9:**
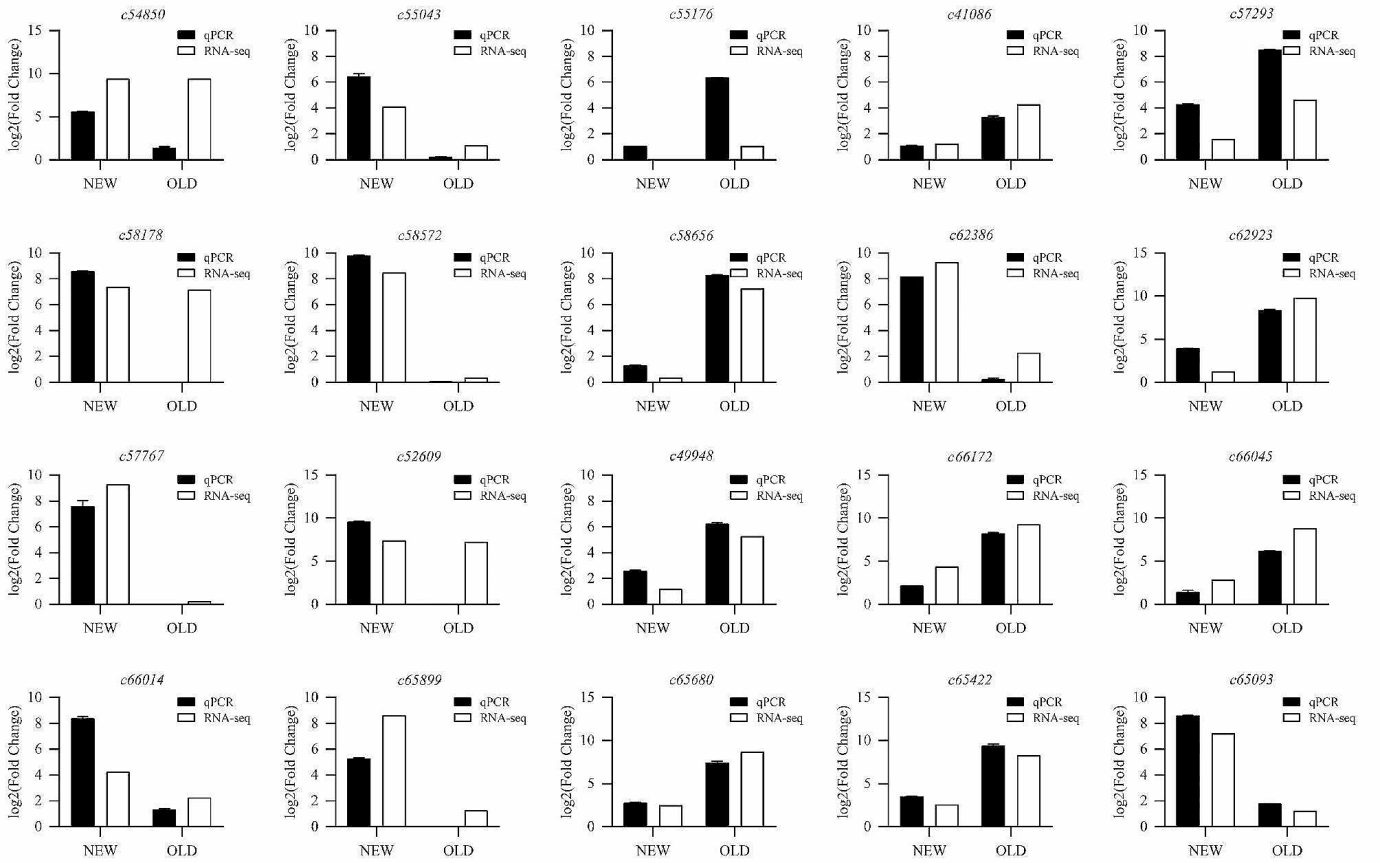
Expression analysis of related genes determined by qPCR and RNA-seq. The abscissa is the sample name, OLD stands for old leaf, NEW stands for new leaf. The ordinate is the log value of relative gene expression, the black is the gene expression result of qPCR in three biological replicates, the white is the FPKM log value of the gene in RNA-seq, and the white bar graph is the average value of NEW and OLD target genes for drawing, so there is no error bar

To further verify the expression patterns of biosynthetic genes revealed by RNA sequencing, 24 DEGs (Table S7) were selected and their expression levels in new and old leaves were detected by RT-qPCR. As shown in Fig. 9, the results of RT-qPCR were consistent with the expression data obtained by RNA-Seq, which represented that the expression of related genes in the transcriptome data could be in line with the actual expression trend of the target gene in each sample, and verified the scientific nature of the transcription data.

## Discussion

In this study, by comparing the contents of flavonoids and alkaloids in the new and old leaves of *M. tetramera*, it was preliminarily shown that the content of total flavonoids and total alkaloids in the old leaves was higher than that in the new leaves. In addition, the content of aromatic amino acids in the new and old leaves was further detected, and tyrosine and tryptophan were significantly more accumulated in the old leaves. The results indicated that there must be significant differences in the synthetic regulatory pathways of metabolites in leaves during the growth process of *M. tetramera*, which might be caused by the differential expression of specific transcriptional regulatory genes. However, the multi-omics research of *M. tetramera* is still in its initial stage. Therefore, this study systematically carried out a conjoint analysis of its transcriptome and metabolome based on the physical and chemical differences between the old leaves and the new leaves of *M. tetramera*. Several different metabolites were identified by analyzing the metabolome of new and old leaves of *M. tetramera*. To understand the synthesis mechanism of DAMs, the transcriptome of the new and old leaves of *M. tetramera* was further determined, and the transcriptome and metabolome were jointly analyzed, focusing on the analysis of the synthesis mechanism of flavonoids and alkaloids that affect the accumulation of medicinal ingredients in the new and old leaves.

### Accumulation mechanism of flavonoid compounds in leaves of *M. tetramera*

Flavonoids are the main phenolic compounds, and the basic structure of flavonoid compounds is composed of two aromatic rings (A, B) and a heterocyclic ring(C), which constitute the basic skeleton structure of C6-C3-C6. The synthesis of flavonoid compounds has three stages. The first stage is the synthesis of precursors. The synthesis of precursors for flavonoid compounds is carried out by two different pathways: the acetic acid pathway and the shikimate pathway, the three malonyl-CoA molecules produced by glucose conversion synthesize ring A through the acetic acid pathway, and the 4-coumaroyl-CoA generated by phenylalanine through the shikimate pathway synthesizes ring B. In the second stage, flavonoids are synthesized. Ring A and ring B condense to form chalcone, which is isomerized to naringenin under the catalysis of chalcone isomerase (CHI), and they are very important intermediates in the flavonoid synthesis pathway. The third stage is the glycosylation of flavonoids to form various glycosides [4, 26]. In citrus plants, the precursor of flavonoids is p-coumaroyl-CoA, which was derived from the phenylpropanoid pathway. Phenylalanine is the most primitive precursor, phenylalanine which eliminates amines through phenylalanine ammonia-lyase (PAL), and then under the catalysis of trans-cinnamate 4-monooxygenase (CH4), CH4 is hydroxylated to form p-coumarate, which is further acylated to p-coumaroyl-CoA by 4-coumarate–CoA ligase (4CL) and acetyl-CoA, p-coumaroyl-CoA served as the central intermediate of the biosynthetic pathway and is further transformed to a different flavonoid compound [27]. In this study, a CYP73A enzyme, rather than CH4, was detected and its substrate was trans-cinnamate (Fig. 6). Multiple genes involved in flavonoid biosynthesis, CHS1, CHI3, F3H, DFR, and ANS were identified in *Angelica sinensis*, which in turn converted 4-coumaroyl-CoA to naringenin chalcone, naringenin, dihydrokaempferol, leucoanthocyanidin, and pelargonidin [28]. Shoeva O Y et al. demonstrated the regulatory role of the Ant2 gene in anthocyanin biosynthesis in barley grain pericarp [29]. Liu, Y. et al. speculated that structural genes such as 4CL1, CYP73A, and CYP75B1 in *Syringa oblata* Lindl. were involved in the flavonoid biosynthesis pathway and significantly up-regulated rutin biosynthesis [30]. DFRs, LARs, and ANRs in *Ginkgo biloba* were also significantly associated with flavonoid accumulation. With the elevation of plant habitats, these genes’ expression up-regulated, resulting in flavonoid accumulation [31]. In addition, multiple genes related to flavonoid biosynthesis were identified in Michelia crassipes and Canarium album [32, 33]. In this study, the key genes detected in the phenylpropanoid pathway also include CCR, HCT, CAD, F5H, etc. The metabolites p-Coumaryl alcohol (meta_137) and Sinapyl alcohol (meta_306) were higher in OLD than in NEW (Fig. 6). The up-regulated expression of CCR might promote the accumulation of p-Coumaryl alcohol, and the up-regulated expression of F5H might indirectly promote the accumulation of Sinapyl alcohol. In the flavonoid synthesis pathway, the key genes detected include CHI, CHS, DFR, ANR, PGT1, etc., which are significantly correlated with flavonoid synthesis. The significant accumulation of phloretin might inhibit the expression of CHS. In addition, flavonoid synthesis was also regulated by other pathways. Leonardo Perez de Souza et al. also confirmed that the acetate pathway affects the level of flavonoid and lipid synthesis, and the absence of the acetate pathway would lead to a decrease in flavonoid content [34]. KEGG enrichment analysis showed that DEGs were enriched in Plant hormone signal transduction, plant-pathogen interaction, and Circadian rhythm-plant pathways. These three important pathways may have an effect on the accumulation of flavonoids and alkaloids. Plant hormones play an important role in plant growth and development and stress response. Through plant hormone signaling transduction pathways, plants can regulate the synthesis of flavonoids and alkaloids. Under stressful conditions, plants may synthesize specific secondary metabolites, including flavonoids and alkaloids, in response to the invasion of various pathogens that may be present in the growing environment. The physiological state and metabolic activities of plants will change under the diurnal variation, and the synthesis of flavonoids and alkaloids may be regulated by the circadian rhythm. Bing He et al. found in the study of *Ginkgo biloba* that stilbenes and flavonoids share the same precursor synthesis pathway, and flavonoids might be used as substrate supplements for stilbenes to strengthen plant resistance to the environment and diseases [35]. Exogenous stimulation could also significantly affect the expression levels of flavonoids, etc. For example, exogenous application of phenylalanine could increase the total content of flavonoids and promote the expression of some flavonoid biosynthesis-related genes in *chayote* (*Sechium edulel*) fruits [36]. Under the induction of salicylic acid, the expression levels of flavonoid-related genes in wheat were significantly increased [37], such as under JA (Jasmonic acid) treatment, the expression of anthocyanin biosynthesis genes *DFR*, *LDOX* and *UF3GT* was promoted, thereby regulating anthocyanin biosynthesis in *Arabidopsis thaliana* [38]. Therefore, in the follow-up study, we can also determine the expression of the above-mentioned flavonoid synthesis regulatory genes of *M. tetramera* through the action of gradient exogenous hormones, and explore whether the optimal exogenous hormones and concentrations that can effectively improve the flavonoid accumulation during its cultivation.

### The accumulation mechanism of alkaloid compounds in the leaves of *M. tetramera*

Alkaloids are a special class of metabolites derived from amino acids. The synthesis of alkaloids first requires the accumulation of amine and aldehyde precursors. The amine precursors for complex alkaloids are polyamines from lysine, arginine, as well as ornithine and aromatic amines from tryptophan and tyrosine. The aldehyde precursors must accumulate to a certain concentration and be proximate to the amine precursors before the next stage of alkaloid biosynthesis can proceed, leading to the formation of reactive imine intermediates. For polyamine-derived alkaloids, such aldehydes are amino aldehydes formed by oxidative deamination of the terminal amines on the polyamine precursors. In the case of aromatic amine-derived alkaloids, parts of the aldehydes are present on different molecules, usually as secondary metabolites [10, 39]. Among these, Tropane Alkaloids are converted from ornithine to N-methyl-1-pyrrolinium cation through consecutive enzymatic reactions mediated by ornithine decarboxylase (ODC), putrescine *N*-methyltransferase (PMT), and *N*-methylputrescine oxidase (MPO), and followed by spontaneous cyclization. Pyrrolidine ketide synthase (PYKS) catalyzes the condensation of imine–ketide compounds in constructing the tropane skeleton, which is then catalyzed by the cytochrome P450 enzyme of the CYP82M subfamily (AbCYP82M3 in *Atropa belladonna*) to form a second ring, producing tropinone [40, 41]. Norcoclaurine synthase (NCS), Deacetylipecoside synthase, and Strictosidine synthase (STR) are the enzymes involved in alkaloid synthesis in plants [39]. In *M. tetramera*, c67211.graph_c0 and c61580.graph_c0 are annotated as NCS and STR, respectively, and they were significantly expressed in old leaves, beneficial to alkaloid synthesis. Additionally, since alkaloids were nitrogen-containing amino acid derivatives, their synthesis also required the action of transaminases. In this study, 44 genes were annotated as transaminases. They might fulfill the role of the afore mentioned transaminases, contributing to the continuous accumulation of alkaloids in the leaves of *M. tetramera*, with 8 genes significantly highly expressed in old leaves.

### The mechanism of flavonoid and alkaloid synthesis in leaves of *M. tetramera* reveals the research significance of this species

Among the DAMs related to flavonoid and alkaloid synthesis, some metabolites significantly impact plant growth and stress resistance. In this study, the significant accumulation of Sinapoyl malate (meta_625) in old leaves can protect leaves from ultraviolet radiation [42]. Conversely, a reduction in Sinapoyl malate levels may render plants highly sensitive to continuous light exposure [43]. Sinapyl alcohol (meta_306), a precursors in plant lignin synthesis, contributes to the structural integrity of plants. Phloretin (meta_414) exhibits antibacterial, anti-inflammatory [44, 45], antioxidant activity, anti-cancer activity [46], and anti-aging effects. Similarly, Isoquercitrin (meta_812) is also a bioactive flavonoid compound with antioxidant and anti-inflammatory activities [47], capable of inducing apoptosis of cancer cells via the AMPK/mTOR/p70S6K pathway [48] and demonstrating cytotoxic effects [49]. Isoflavonoids not only participate in carbohydrate metabolism, regulate blood sugar, and is able to reduce glycosuria complications [50] but also have antioxidant and antitumor effects [8, 51]. L-Tyrosine (meta_231) containing highly active α-amino, α-carboxylic, and phenolic hydroxyl groups, can produce derivatives through a series of derivatization reactions. At the same time, L-Tyrosine supplementation may assist the elderly in maintaining core body temperature in cold environments [52]. Piperine (meta_453) has a variety of medical effects such as antioxidant, anti-inflammatory, anti-aging and neuroprotection [53]. Citric Acid (CA) can improve plant abiotic stress tolerance. Application of CA can promote the increase of chlorophyll content, improve antioxidant resistance, alleviate heavy metal stress, etc.., [50] and it can play a role in blood anticoagulation and enhance carbon dioxide scavenging [54]. The elevated levels of these DAMs in the old leaves of *M. tetramera* not only suggest a higher content of medicinal substances compared to new leaves but also indicate superior disease resistance and environmental stress tolerance. This might be one of the mechanisms behind the evergreen state of the old leaves.

### The importance of transcription factors in regulating metabolite accumulation in plants

In the process of biosynthesis, besides the structural genes encoding enzymes, transcription factors also play a crucial role in regulation. Pu, YT et al. annotated an R2R3-MYB transcription factor by transcriptome sequencing assembly in the study of *Chayote* (*Sechium edulel*) fruits, which was found to be key in regulating flavonoid synthesis in *Sechium edulel* [55]. Previous studies have indicated that the same transcription factor can exhibit different regulatory modes across species [27]. MYB is an important transcription factor in anthocyanin biosynthesis. EsMYB90 is pivotal in the biosynthesis of anthocyanins and flavonoids in tobacco, regulating the expression of flavonoid biosynthesis genes and promoting the accumulation of flavonoid compounds [56]. However, the GbMYBR1 transcription factor in *Ginkgo biloba* has been identified as a regulatory factor that promotes flavonoids synthesis in *Ginkgo biloba* leaves. Its overexpression in *Arabidopsis thaliana* led to leaf growth retardation, a decrease in lignin content, and a significant decrease in anthocyanin and flavonol content [21], indicating that specific transcription factors in different plants significantly influence flavonoids synthesis. This may be constrained by transcription factors interaction, such as those within MYB-BHLH-WRD (MBW) complexes, which constitute an essential regulatory mechanism affecting flavonoid synthesis in plants, with varying mechanisms for regulating transcriptional activity [22]. Studies on the transcriptional regulation of alkaloids have shown that BHLH, ERF, and WRKY transcription factors also induce alkaloid synthesis [57–59]. The plant hormone jasmonate (JA) can trigger the expression of some transcription factor genes, thereby inducing defense-related genes to produce defense proteins and secondary metabolites [60]. Moreover, the synthesis of plant metabolites is influenced not only by the growth stage and tissue site but also by environmental factors such as light and altitude [7, 13, 61]. In this study, we annotated that 3 MYB transcription factors and 12 bHLH transcription factors (Fig. 5) from the old leaves of *M. tetramera* as significantly up-regulated, suggesting a mechanism that may promote the synthesis and regulation of flavonoids and alkaloids during the development of *M. tetramera* leaves, warranting further experimental verification.

*M. tetramera* is rich in bioactive flavonoids and carbazole alkaloids. Currently, a variety of active ingredients have been identified and have been used in biomedicine and other aspects. Yet, the biosynthesis mechanism of flavonoids and alkaloids in *M. tetramera* remain elusive. This study conducted transcriptome and metabolite analysis of leaves under different growth cycles to elucidate the flavonoid and alkaloid components in *M. tetramera*, identifying genes involved in their biosynthesis. This research lays the groundwork for further exploration of these compounds’ biosynthetic pathways in M. tetramera. However, the limited number of developmental time points examined in this study restricts our understanding of the link between transcriptional genes and flavonoid and alkaloid biosynthesis. Therefore, the trend analysis of genes and metabolites related to flavonoid biosynthesis in old leaves has not been performed. The research team plans to address and enhance these aspects in future studies. A large number of secondary metabolic substances produced by plants enable plants to resist stress and promote their growth in response to environmental changes. In general, most of the metabolites were higher in the old leaves have anti-oxidant, anti-aging, and stress resistance effects, which may be one of the reasons why the old leaves can still maintain a certain luster and texture. In conclusion, through the conjoint analysis of the metabolome and transcriptome of *M. tetramera*, the DAMs between new and old leaves were determined, and the genes related to the synthesis of the main flavonoids and alkaloids of *M. tetramera* were identified and RT-qPCR was carried out to verify. These findings not only contribute to understanding the medicinal value of *M. tetramera*, but also provide a foundation for developing artificial cultivation techniques and formulating high-yield harvesting strategies for this plant.

## Conclusions

*M. tetramera* is a traditional Chinese herbal medicine, and the identification of its medicinal components is still an important research direction in the modern pharmaceutical field. In this study, through combined transcriptome and metabolomics analysis, the differences in transcriptome and metabolites between old and new leaves of *M. tetramera* were compared, and the network regulation of flavonoids and alkaloids of *M. tetramera* was preliminarily constructed. The analysis and annotation showed that 125 transcriptional genes may have regulatory effects on the metabolism of flavonoids and 48 transcriptional genes that regulate alkaloid synthesis, including 15 MYB and 32 bHLH transcription factors, which regulate flavonoid substances and alkaloid synthesis. The accumulation of flavonoids and alkaloids may be correlated with 3 upregulated MYB and 11 upregulated bHLH transcription factors in the old leaves. They positively regulate the expression of PAL, CYP73A, 4-coumarate-CoA sinapyl, 4CL, CCR, F5H, CHS, DFR, ANR, PGT1, and other enzymes in the transcription network of flavonoids and alkaloids, resulting in the accumulation of flavonoids in old leaves. The positive regulation of tyrosine/DOPA decarboxylase 5, probable carboxylesterase 15, probable carboxylesterase 16, carboxylesterase 1-like, tropinone reductase, homolog isoform X1 and other enzymes resulted in the accumulation of alkaloids in old leaves. In the future, we will further validate the functions of the identified transcription factors and key enzymes of metabolic pathways. These genes were knocked out or overexpressed by genetic engineering or CRISPR/Cas9 technology to directly observe their effects on flavonoid and alkaloid content in *M. tetramera*. Whole genome sequencing and epigenetic analysis will be conducted to gain a more comprehensive understanding of its genetic background and regulatory mechanisms of gene expression, which will help reveal the genetic basis and regulatory network of the synthesis of medicinal components of *M. tetramera*.

## Methods

### Plant materials

In this study, the seeds of *M. tetramera* were collected from Duyang Forest Farm (23.974182 ‘N, 107.65657’ E) in Dahua County, Guangxi, China, and culture in a greenhouse in a suburb of Changsha City, Hunan Province. The plant was identified by botanist Chengchiu Huang as a member of the genus Murraya in the family Rutaceae. The leaves with a growth cycle of 1 year and 6 months were classified as OLD leaves and those with a growth cycle of 1–2 months were classified as NEW leaves for biological sampling. The leaves were collected in April 2021 and sent to BioMarker Biological Company for metabolomics and transcriptome analysis. And a part of samples were immediately frozen in liquid nitrogen and stored at -80 °C for the determination of total flavonoids and alkaloids and Aromatic amino acid content. To ensure the quality of omics data, leaf samples from random plants were used for metabolomics and transcriptome samples, including three biological replicates (NEW1, NEW2, NEW3, OLD1, OLD2, OLD3) for transcriptome sequencing and four biological replicates for metabolomics samples, NEW4 and OLD4 samples were added based on transcriptome sampling.

### Determination of total alkaloids and flavonoids

Determination of total alkaloid content: 0.5 g of *M. tetramera* leaf samples were ground in liquid nitrogen, sonicated with 80% ethanol for 1 h, after filtering and washing the filter residue, fixed volume to 25 mL. Weighed 5 g of macroporous resin, soaked it in 95% ethanol for 24 h, rinsed it with deionized water until alcohol-free, and the sample was placed in the treated ion exchange column for adsorption at a rate of 2 BV. Then 20 mL of deionized water was used to clean the resin at a rate of 3 BV. After cleaning, 25 mL of 80% ethanol was used for desorption with a speed of 2 BV, and the liquid was collected. The absorbance value of the sample was determined by TU-1901 UV -Vis Spectrometer (Beijing Persee General Instrument Co., Ltd.) at a wavelength of 203 nm, 80% ethanol was used as blank control, and the standard was Dendrobine. The total alkaloid content was calculated from the standard curve.

Total flavonoid content determination: The sample was dried to a constant weight at 70 °C and ground to a fine powder. After a 40-mesh screen, a 0.5 g sample was accurately weighed and transferred to 4 mL 50% ethanol solution in a water bath at 60 °C for 60 min for extraction, shaken once every 10 min, filtered under pressure while hot, and washed twice with 50% ethanol solution. The volume was fixed to 25 mL. Then 0.5 mL of sample solution was added to a brown 25 mL volumetric flask, 1.0 mL of 5% NaNO_2_ reagent was added, shaken upside down, left in the dark for 6 min, and then 1.0 mL of 10% Al (NO_3_)_3_ reagent was added. The reaction was left to stand in the dark for 6 min after the reaction was reversed and shaken, and then 10 mL of 1 mol/L NaOH solution was added to terminate the reaction. Finally, the reaction solution was fixed to the scale with deionized water. A standard curve was made using rutin as a standard to calculate the flavonoid content and three biological replicates were performed for each sample. All the above reagents were purchased from Hunan Hemiao Biotechnology Co., LTD., China, and the specifications were all analytical pure reagents.

Aromatic amino acid content determination: After accurately weighing 0.5 g of fresh leaves and grinding them in liquid nitrogen, with 0.1 mol/L sodium hydroxide solution was extracted as solvent. L-phenylalanine ammoniase (PAL)elisa kit, tryptophan (TRP)ELISA kit, and tyrosine (TyR)ELISA kit of Aibo Biotech Co., LTD (Shanghai, China) were used to detect the content of phenylalanine, tryptophan and tyrosine in new and old leaves of *M. tetramera*.

### Methods for metabolite extraction and analysis

Sample preparation: Freeze-dried leaves of *M. tetramera* were ground in a grinder (Mixer Mill MM 400, Retsch, Haan, Germany) for 1.5 min at 30 Hz to a powder form, 100 mg of the powder was weighed and dissolved in 1.2 mL of 70% methanol solution for extraction. The samples were vortexed every 30 min for 30 s for a total of 6 times, and then the samples were extracted at 4 °C for 12 h. The samples were then centrifuged at 12,000 r/min for 10 min. The supernatant was filtered through 0.22 μm microporous filters for UPLC-MS/MS analysis.

UPLC Analysis: Data analysis was performed by using UPLC (SHIMADZU Nexera X2, Shimadzu, Japan) on AgilentSB-C18 column,2.1 mm×100 mm, 1.8 μm (Agilent, New York, USA) at temperature 40 °C. Gradient elution was carried out with mobile phases A and B. The mobile phase A was ultrapure water added with 0.1% formic acid, and the mobile phase B was acetonitrile added with 0.1% formic acid. After column equilibration, the injection volume of each sample was 4 µl. The elution gradient showed that the proportion of phase B was 5% at 0.00 min, increased linearly to 95% at 9.00 min, and maintained at 95% for 1 min. At 10.00-11.10 min, the proportion of phase B decreased to 5% and equilibrated at 5% until 14 min. The flow rate was 0.35 ml/min. (Formic acid and acetonitrile were purchased from Hunan Hemiao Biotechnology Co., LTD., Changsha, Hunan, China, and the specifications were chromatographic grade.)

MS Analysis: The UPLC effluent was analyzed using MS system (Applied Biosystems 4500 QTRAP, http://www.appliedbiosystems.com.cn/) with ESI-Turbo ion spray interface (AB SCIEX, Boston, MA, USA). The operating parameters were as follows: ESI source temperature 550 °C, positive ion spray voltage 5.5 kV, negative ion spray voltage 4.5 kV, curtain gas 25 psi, and collision-activated dissociation device 5 pis. Triple quadrupole scans were acquired as MRM experiments with optimized de-clustering potential and collision energy CE for each individual multiple reaction monitoring (MRM) transition.

The total ion flow graphs of all samples in positive and negative ion modes were subjected to baseline filtering, peak identification, integration, retention time correction, peak alignment and normalization by Progenesis QI to obtain the data matrix of retention time, mass-charge ratio (m/z) and peak intensity. After the data matrix was imported into SIMCA software, Principal component analysis (PCA) and orthogonal projections to latent structure-discriminant analysis (PLS-DA) were performed. Metabolites are identified using internal and public databases (MassBank, KNApSAcK, HMDB, MoTo DB and METLIN) by comparing mass-charge ratio, retention time and fragmentation patterns to standards. DAMs in different samples were screened using the criteria of VIP value > 1, FC ≥ 1 and P value < 0.05. Metabolic pathways of DAMs were also investigated using the KEGG database (KEGG PATHWAY Database (genome.jp)).

### Total RNA extraction and transcriptome sequencing

Total RNA was extracted from 0.5 g fresh leaves using a Tiagen DP441 kit (Tiagen Biochemical Technology Co., LTD., Beijing, China), and the concentration of extracted RNA was detected using Nanodrop2000(manufacturer: Thermofly, model: Nanodrop2000). Agient2100(Agilent Technologies Inc., California, United States) and LabChip GX (manufacturer platinum, model Platinum Elmer LabChip GX) were used for integrity testing. Qualified RNA was used for transcriptome sequencing. The original transcriptome data have been uploaded to the National Center for Biotechnology Information (NCBI, National Library of Medicine, Bethesda, MD., USA) database (PRJNA983954).

A transcriptome library was constructed according to the Botanics research method [33], and based on Sequencing by Synthesis (SBS) technology, six leaf cDNA libraries were constructed using Illumina NovaSeq 6000 platform (Illumina, San Diego, CA., USA) high-throughput sequencing platform. A large number of Raw Data were obtained, then quality control was carried out, sequencing adapter and primer sequences in Reads were truncated, and low-quality Data were filtered to obtain Clean Data. Trinity was used for assembly to obtain unigenes [62]. The function of unigenes was annotated using the BLAST program, including NR(https://blast.ncbi.nlm.nih.gov/Blast.cgi, accessed on 1 January 2022), Swiss-Prot(https://www.uniprot.org/, accessed on 1 January 2022), GO(http://geneontology.org/, Accessed on 1 January 2022) and COG (https://www.ncbi.nlm.nih.gov/COG/, Accessed on 1 January 2022), KOG (https://www.ncbi.nlm.nih.gov/KOG/, accessed on 1 January 2022), Pfam(http://pfam.xfam.org/, accessed on 1 January 2022) and KEGG(https://www.kegg.jp/, accessed on 1 January 2022)., and the differential expression analysis between sample groups was performed in line DESeq2 [63]to compare the ratio of FPKM between samples (FC, fold change), and false discovery rate (FDR). The DEGs between the new leaf and the old leaf were screened with FC ≥ 2 and FDR < 0.01. The transcription factors in DEGs were screened by NR annotation.

### Relative expression analyses of selected genes

RNA was extracted from new and old leaves of *M. tetramera*, and Real-Time Quantitative PCR was performed after reverse transcription. The CFX Connect™ fluorescent quantitative PCR Assay System (Bio-Rad Laboratories Inc. Hercules, CA, United States) was used to perform the amplification cycle according to the TaKaRa TB Green® Premix Ex Taq™ II FAST qPCR Kit(Takara Bio Inc., Tokyo, Japan), using a 20 µl reaction system. GAPDH (aldehyde-3-phosphate dehydrogenase) was used as the internal reference gene, and the gene expression data were presented in the form of relative expression. The relative expressions of each sample and each group were calculated by 2^−△△Ct^. Each sample in biological repeated three times.

### Statistical analysis

In this study, three biological replicates were performed for all experiments, and SPSS Statistics 23 was used for T-test, with *p* < 0.05 as the basis for statistical differences, and draw the graph through origin 2018.

### Correlation analysis between metabolite spectrum and transcripts

The DEGs and DAMs were screened out by conditions, and the correlation between all genes and metabolites was calculated for each group based on the Pearson correlation method. Before the correlation was calculated, the z-value transformation method was used for data pre-processing, then the screening is carried out according to the Correlation Coefficient (CC) and the p-value of the correlation, and the screening threshold is:|CC|>0.80 and CCP < 0.05. The KEGG enrichment pathway was constructed and the interaction network between DEGS and DAMs was visualized using an AI mapping tool (Adobe Illustrator 2020).

### Electronic supplementary material

Below is the link to the electronic supplementary material.

Supplementary Material 1

## Data Availability

The datasets generated and analyzed during the current study are available in the supplementary information file. The original transcriptome data have been uploaded to the National Center for Biotechnology Information (NCBI, National Library of Medicine, Bethesda, MD., USA) database (PRJNA983954).
