# Supplementary material for: Integrated metabolomic and transcriptomic analysis reveals the regulatory mechanisms of flavonoid and alkaloid biosynthesis in the new and old leaves of Murraya tetramera Huang
Source: BMC Plant Biol. 2024 Jun 5;24:499. doi: 10.1186/s12870-024-05066-9 (PMC11151518; doi:10.1186/s12870-024-05066-9)
Supplement: Supplementary file 1 — Supplementary Material 1 [file 12870_2024_5066_MOESM1_ESM.docx]

**Figure S1.** KEGG Classification Map of Differentially Expressed Genes between New Leaves and Old Leaves of *M. tetramera* Huang

**
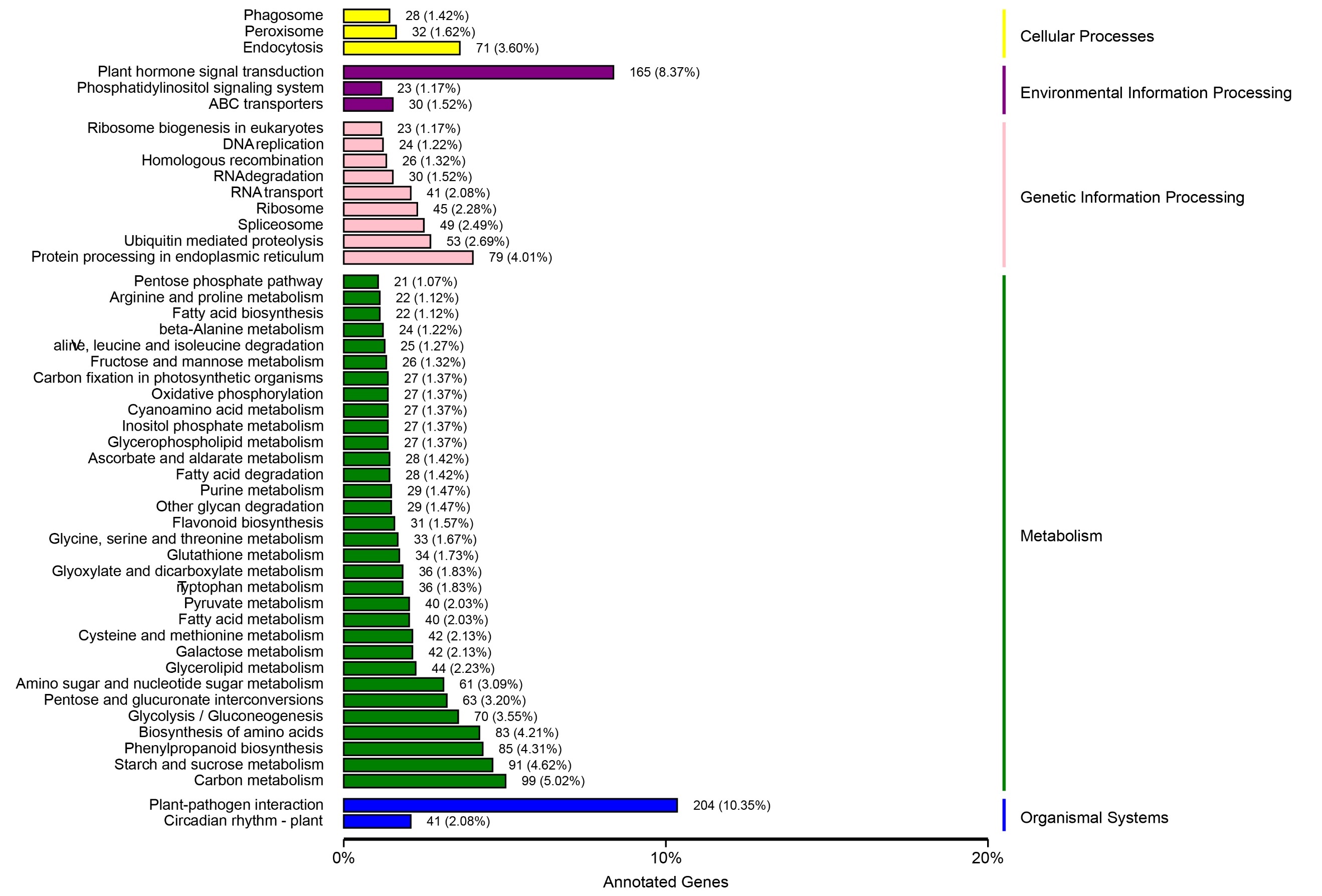
**

**Figure S2.** Column diagram of co-enrichment of differential metabolites and differential genes between new leaves and old leaves of *M. tetramera* Huang


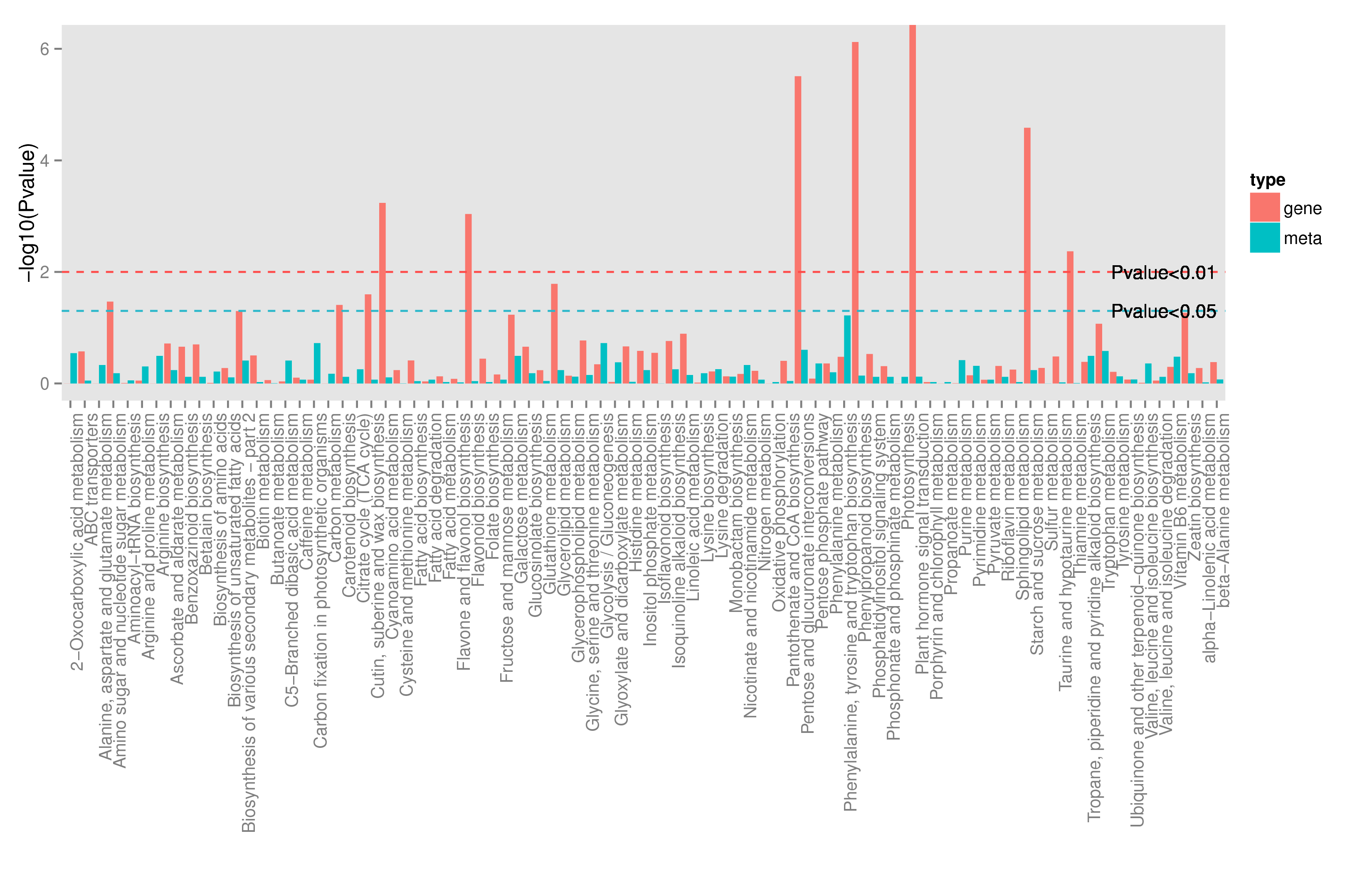


**Table S1.** Metabolite classification statistics

| classification | Number | classification | Number |
| --- | --- | --- | --- |
| Carboxylic acids and derivatives | 64 | Carboximidic acids and derivatives | 2 |
| Fatty Acyls | 49 | Cinnamyl alcohols | 2 |
| Organooxygen compounds | 43 | Diazines | 2 |
| Benzene and substituted derivatives | 15 | Keto acids and derivatives | 2 |
| Flavonoids | 13 | Lignan glycosides | 2 |
| Organonitrogen compounds | 12 | Linear 1,3-diarylpropanoids | 2 |
| Phenols | 9 | Organic phosphoric acids and derivatives | 2 |
| Prenol lipids | 9 | Phenylpropanoic acids | 2 |
| Indoles and derivatives | 8 | Steroids and steroid derivatives | 2 |
| Cinnamic acids and derivatives | 7 | (5'->5')-dinucleotides | 1 |
| Purine nucleosides | 7 | 3,4-dihydrocoumarins | 1 |
| Purine nucleotides | 7 | 5'-deoxyribonucleosides | 1 |
| Coumarins and derivatives | 6 | Benzopyrans | 1 |
| Pyrimidine nucleosides | 5 | Biotin and derivatives | 1 |
| Hydroxy acids and derivatives | 4 | Flavin nucleotides | 1 |
| Pyridines and derivatives | 4 | Glycerophospholipids· | 1 |
| Cinnamaldehydes | 3 | Isoquinolines and derivatives | 1 |
| Glycerolipids | 3 | Organic carbonic acids and derivatives | 1 |
| Imidazopyrimidines | 3 | Piperidines | 1 |
| Isoflavonoids | 3 | Quinolines and derivatives | 1 |
| Pyrimidine nucleotides | 3 | Ribonucleoside 3'-phosphates | 1 |
| Azoles | 2 | Tropane alkaloids | 1 |

## Table S2 Summary of sequencing quality. BMK-ID: sample analysis number; Read Number: the total number of pair-end Reads in Clean Data; Base Number: the total base number of Clean Data; GC Content: The percentage of G and C bases in the total base in the Clean Data; %≥Q30: The percentage of bases with Clean Data quality values greater than or equal to 30.

| BMK-ID | Read Number | Base Number | GC Content | %≥Q30 |
| --- | --- | --- | --- | --- |
| NEW1 | 24,731,077 | 7,399,881,072 | 44.61% | 94.10% |
| NEW2 | 24,525,168 | 7,334,527,506 | 44.72% | 94.45% |
| NEW3 | 24,919,124 | 7,454,057,738 | 44.61% | 94.71% |
| OLD1 | 24,896,621 | 7,446,766,612 | 44.43% | 94.03% |
| OLD2 | 24,570,777 | 7,350,066,542 | 44.38% | 94.38% |
| OLD3 | 24,613,065 | 7,361,087,892 | 44.39% | 94.31% |

**Table S3** Length distribution of unigenes and transcripts

| Length Range | Transcript | Unigene |
| --- | --- | --- |
| 300-500 | 20,286(5.49%) | 13,947(35.77%) |
| 500-1000 | 30,965(8.37%) | 9,895(25.38%) |
| 1000-2000 | 81,641(22.08%) | 6,388(16.38%) |
| 2000+ | 236,917(64.06%) | 8,759(22.47%) |
| Total Number | 369,809 | 38,989 |
| Total Length | 1,036,373,416 | 51,782,720 |
| N50 Length | 3,546 | 2,420 |
| Mean Length | 2802.46 | 1328.14 |

**Table S4** Unigenes annotated to the nine databases

| #Anno_Database | Annotated_Number | 300<=length<1000 | length>=1000 |
| --- | --- | --- | --- |
| COG_Annotation | 7,773 | 2,890 | 4,883 |
| GO_Annotation | 20,376 | 8,318 | 12,057 |
| KEGG_Annotation | 16,276 | 6,084 | 10,192 |
| KOG_Annotation | 14,266 | 5,756 | 8,510 |
| Pfam_Annotation | 18,629 | 7,138 | 11,491 |
| Swissprot_Annotation | 15,358 | 5,005 | 10,353 |
| TrEMBL_Annotation | 23,655 | 9,557 | 14,098 |
| eggNOG_Annotation | 19,351 | 7,139 | 12,212 |
| nr_Annotation | 25,551 | 11,342 | 14,209 |
| All_Annotated | 26,587 | 12,302 | 14,284 |

**Table S5.** List of GO enrichment of DEGs during *M. tetramera* Huang

| #GO_classify1 | GO_classify2 | DE |
| --- | --- | --- |
| #Total_gene |  | 4777 |
| cellular component | extracellular region | 96 |
| cellular component | cell | 1696 |
| cellular component | nucleoid | 1 |
| cellular component | membrane | 1692 |
| cellular component | virion | 1 |
| cellular component | cell junction | 56 |
| cellular component | membrane-enclosed lumen | 107 |
| cellular component | macromolecular complex | 318 |
| cellular component | organelle | 1235 |
| cellular component | other organism | 54 |
| cellular component | other organism part | 54 |
| cellular component | extracellular region part | 10 |
| cellular component | organelle part | 447 |
| cellular component | virion part | 1 |
| cellular component | membrane part | 1519 |
| cellular component | synapse part | 1 |
| cellular component | cell part | 1696 |
| cellular component | synapse | 1 |
| cellular component | symplast | 52 |
| cellular component | supramolecular complex | 67 |
| molecular function | transcription factor activity, protein binding | 18 |
| molecular function | nucleic acid binding transcription factor activity | 175 |
| molecular function | catalytic activity | 2183 |
| molecular function | signal transducer activity | 59 |
| molecular function | structural molecule activity | 69 |
| molecular function | transporter activity | 319 |
| molecular function | binding | 2328 |
| molecular function | electron carrier activity | 45 |
| molecular function | antioxidant activity | 37 |
| molecular function | metallochaperone activity | 1 |
| molecular function | protein tag | 1 |
| molecular function | nutrient reservoir activity | 4 |
| molecular function | molecular transducer activity | 56 |
| molecular function | molecular function regulator | 88 |
| biological process | reproduction | 93 |
| biological process | immune system process | 18 |
| biological process | behavior | 2 |
| biological process | metabolic process | 2062 |
| biological process | cellular process | 1946 |
| biological process | reproductive process | 93 |
| biological process | biological adhesion | 1 |
| biological process | signaling | 212 |
| biological process | multicellular organismal process | 156 |
| biological process | developmental process | 172 |
| biological process | growth | 18 |
| biological process | locomotion | 8 |
| biological process | single-organism process | 1484 |
| biological process | rhythmic process | 12 |
| biological process | response to stimulus | 573 |
| biological process | localization | 492 |
| biological process | multi-organism process | 93 |
| biological process | biological regulation | 857 |
| biological process | cellular component organization or biogenesis | 317 |
| biological process | cell aggregation | 1 |
| biological process | detoxification | 44 |

**Table S6.** List of KEGG pathway enrichment of DEGs and DEMs during *M. tetramera* Huang

| #Kegg_pathway | ko_id | Gene | Metabolite |
| --- | --- | --- | --- |
| Glycolysis / Gluconeogenesis | ko00010 | 70 | 6 |
| Citrate cycle (TCA cycle) | ko00020 | 15 | 5 |
| Pentose phosphate pathway | ko00030 | 21 | 3 |
| Pentose and glucuronate interconversions | ko00040 | 63 | 5 |
| Fructose and mannose metabolism | ko00051 | 26 | 2 |
| Galactose metabolism | ko00052 | 42 | 8 |
| Ascorbate and aldarate metabolism | ko00053 | 28 | 2 |
| Fatty acid biosynthesis | ko00061 | 22 | 3 |
| Fatty acid degradation | ko00071 | 28 | 2 |
| Cutin, suberine and wax biosynthesis | ko00073 | 16 | 2 |
| Ubiquinone and other terpenoid-quinone biosynthesis | ko00130 | 12 | 4 |
| Oxidative phosphorylation | ko00190 | 27 | 1 |
| Photosynthesis | ko00195 | 10 | 1 |
| Arginine biosynthesis | ko00220 | 7 | 8 |
| Purine metabolism | ko00230 | 29 | 14 |
| Caffeine metabolism | ko00232 | 2 | 2 |
| Pyrimidine metabolism | ko00240 | 18 | 9 |
| Alanine, aspartate and glutamate metabolism | ko00250 | 9 | 6 |
| Glycine, serine and threonine metabolism | ko00260 | 33 | 6 |
| Monobactam biosynthesis | ko00261 | 2 | 3 |
| Cysteine and methionine metabolism | ko00270 | 42 | 5 |
| Valine, leucine and isoleucine degradation | ko00280 | 25 | 1 |
| Valine, leucine and isoleucine biosynthesis | ko00290 | 3 | 3 |
| Lysine biosynthesis | ko00300 | 2 | 4 |
| Lysine degradation | ko00310 | 18 | 8 |
| Arginine and proline metabolism | ko00330 | 22 | 12 |
| Histidine metabolism | ko00340 | 14 | 6 |
| Tyrosine metabolism | ko00350 | 19 | 8 |
| Phenylalanine metabolism | ko00360 | 16 | 7 |
| Tryptophan metabolism | ko00380 | 36 | 9 |
| Phenylalanine, tyrosine and tryptophan biosynthesis | ko00400 | 16 | 10 |
| Benzoxazinoid biosynthesis | ko00402 | 4 | 1 |
| beta-Alanine metabolism | ko00410 | 24 | 4 |
| Taurine and hypotaurine metabolism | ko00430 | 3 | 2 |
| Phosphonate and phosphinate metabolism | ko00440 | 2 | 1 |
| Cyanoamino acid metabolism | ko00460 | 27 | 5 |
| Glutathione metabolism | ko00480 | 34 | 5 |
| Starch and sucrose metabolism | ko00500 | 91 | 2 |
| Amino sugar and nucleotide sugar metabolism | ko00520 | 61 | 4 |
| Glycerolipid metabolism | ko00561 | 44 | 2 |
| Inositol phosphate metabolism | ko00562 | 27 | 2 |
| Glycerophospholipid metabolism | ko00564 | 27 | 3 |
| Linoleic acid metabolism | ko00591 | 6 | 6 |
| alpha-Linolenic acid metabolism | ko00592 | 21 | 2 |
| Sphingolipid metabolism | ko00600 | 20 | 1 |
| Pyruvate metabolism | ko00620 | 40 | 2 |
| Glyoxylate and dicarboxylate metabolism | ko00630 | 36 | 10 |
| Propanoate metabolism | ko00640 | 11 | 1 |
| Butanoate metabolism | ko00650 | 9 | 2 |
| C5-Branched dibasic acid metabolism | ko00660 | 1 | 7 |
| Carbon fixation in photosynthetic organisms | ko00710 | 27 | 6 |
| Thiamine metabolism | ko00730 | 19 | 1 |
| Riboflavin metabolism | ko00740 | 7 | 1 |
| Vitamin B6 metabolism | ko00750 | 6 | 4 |
| Nicotinate and nicotinamide metabolism | ko00760 | 8 | 6 |
| Pantothenate and CoA biosynthesis | ko00770 | 15 | 5 |
| Biotin metabolism | ko00780 | 9 | 1 |
| Folate biosynthesis | ko00790 | 12 | 1 |
| Porphyrin and chlorophyll metabolism | ko00860 | 9 | 1 |
| Carotenoid biosynthesis | ko00906 | 19 | 1 |
| Zeatin biosynthesis | ko00908 | 13 | 4 |
| Nitrogen metabolism | ko00910 | 14 | 2 |
| Sulfur metabolism | ko00920 | 13 | 1 |
| Phenylpropanoid biosynthesis | ko00940 | 85 | 11 |
| Flavonoid biosynthesis | ko00941 | 31 | 3 |
| Isoflavonoid biosynthesis | ko00943 | 7 | 1 |
| Flavone and flavonol biosynthesis | ko00944 | 2 | 2 |
| Isoquinoline alkaloid biosynthesis | ko00950 | 17 | 5 |
| Tropane, piperidine and pyridine alkaloid biosynthesis | ko00960 | 13 | 8 |
| Betalain biosynthesis | ko00965 | 6 | 1 |
| Glucosinolate biosynthesis | ko00966 | 4 | 4 |
| Aminoacyl-tRNA biosynthesis | ko00970 | 19 | 10 |
| Biosynthesis of various secondary metabolites - part 2 | ko00998 | 2 | 7 |
| Biosynthesis of unsaturated fatty acids | ko01040 | 19 | 5 |
| Carbon metabolism | ko01200 | 99 | 12 |
| 2-Oxocarboxylic acid metabolism | ko01210 | 14 | 16 |
| Fatty acid metabolism | ko01212 | 40 | 1 |
| Biosynthesis of amino acids | ko01230 | 83 | 25 |
| ABC transporters | ko02010 | 30 | 25 |
| Phosphatidylinositol signaling system | ko04070 | 23 | 1 |
| Plant hormone signal transduction | ko04075 | 165 | 3 |

**Table S7.** The primers of QRT-PCR

| DEGsID | NR-annotation | Primer (5'-3') |
| --- | --- | --- |
| c41086 | probable glutathione S-transferase | F：TGAAACTCAAGGGCGTTCAA  R：AGGATCGTCGGGAAGCAAAG |
| c52609 | hypothetical protein CUMW_249080 | F: ACTCCCTCCTCCACTCAACA  R: AACAGCCATTGGGTGCTAGG |
| c54850 | alpha-L-fucosidase 1-like | F: GCTTGTCAATGCGGCTAAGG  R: TTTGGCGCATTTGCACCTTT |
| c55176 | secoisolariciresinol dehydrogenase isoform X2 | F：ATGCCCAAGAAAACTCCGGT  R：TGTCCATTGCGATGTCACGA |
| c57293 | probable pyridoxal 5'-phosphate synthase subunit PDX1 | F：AATGGACGTCGTTACTCCCG  R：GGGATGGTGACGGACTGTTT |
| c57767 | GDSL esterase/lipase At5g33370 | F：GCCGCAGCATGCTATCTTTG  R：CTGCAGAACTGCAAAGAGCC |
| c58178 | fatty acyl-CoA reductase 3-like isoform X1 | F: ATCTCCCGCGAAGATTTGGG  R: TGAGACCAAGTGCAACGTCA |
| c58572 | secoisolariciresinol dehydrogenase-like isoform X1 | F: AGCCAATGGAGTTGGGACTG  R: CTGCAAGCGTTTGTGGAGTC |
| c58656 | caffeic acid 3-O-methyltransferase | F：CGAACAAGCGAAGCTCGATG  R：TGCCGTTTTGCGTCTCCATA |
| c62386 | hypothetical protein CISIN_1g007610mg | F: TTTCCAGGACTTCGCTGAGG  R: TTTTCCATGCAGGAGGCGAT |
| c62923 | flavin mononucleotide hydrolase 1, chloroplatic isoform X3 | F：AACCTTGGATCTTCCCTGAGC  R：TTGGCATAACGGGTCTGCAT |
| c64897 | coumarin 8-geranyltransferase 1b, chloroplastic-like | F：AAGCCGTATCTGCCACTTGC  R：AGCTGCTCCTGTTAAACCCC |
| c65093 | auxin-induced protein 22D | F: AACACAAGTAGTGGGGTGGC  R：AGGTGCTCCATGCATGCTAA |
| c65680 | hypothetical protein CUMW_020290 | F：CCAGGTCCCCTTCTGGTAGA  R：GAAGGCAGCATTGGACCTCT |
| c65899 | very-long-chain 3-oxoacyl-CoA reductase-like protein At1g24470 | F: AAATGGCGTCCAGTGTAGCA  R: GAGTGAGCCCAATAAGGCGT |
| c66014 | cytochrome P450 98A2 | F: CTCCCTGCACCAAACGGTAA  R: GTTAAGATCGGCGGCTACGA |
| c66045 | probable carotenoid cleavage dioxygenase 4, chloroplastic | F：AGAAAATGGGCACATCCGGT  R：GACATAGAGACCATCGGGCG |
| c66172 | cytochrome c oxidase copper chaperone 2 | F：GTGGACTGCCTCTGCAAGAT  R：TTCTTAGTCTCGGGGCAAGC |
